# Inhibition of DYRK1A and GSK3B induces human β-cell proliferation

**DOI:** 10.1038/ncomms9372

**Published:** 2015-10-26

**Authors:** Weijun Shen, Brandon Taylor, Qihui Jin, Van Nguyen-Tran, Shelly Meeusen, You-Qing Zhang, Anwesh Kamireddy, Austin Swafford, Andrew F. Powers, John Walker, John Lamb, Badry Bursalaya, Michael DiDonato, George Harb, Minhua Qiu, Christophe M. Filippi, Lisa Deaton, Carolina N. Turk, Wilma L. Suarez-Pinzon, Yahu Liu, Xueshi Hao, Tingting Mo, Shanshan Yan, Jing Li, Ann E. Herman, Bernhard J. Hering, Tom Wu, H. Martin Seidel, Peter McNamara, Richard Glynne, Bryan Laffitte

**Affiliations:** 1 Genomics Institute of the Novartis Research Foundation, 10675 John Jay Hopkins Drive, San Diego, California 92121, USA; 2 Department of Surgery and Schulze Diabetes Institute, University of Minnesota, 420 Delaware Street SE, Minneapolis, Minnesota 55455, USA

## Abstract

Insufficient pancreatic β-cell mass or function results in diabetes mellitus. While significant progress has been made in regulating insulin secretion from β-cells in diabetic patients, no pharmacological agents have been described that increase β-cell replication in humans. Here we report aminopyrazine compounds that stimulate robust β-cell proliferation in adult primary islets, most likely as a result of combined inhibition of DYRK1A and GSK3B. Aminopyrazine-treated human islets retain functionality *in vitro* and after transplantation into diabetic mice. Oral dosing of these compounds in diabetic mice induces β-cell proliferation, increases β-cell mass and insulin content, and improves glycaemic control. Biochemical, genetic and cell biology data point to Dyrk1a as the key molecular target. This study supports the feasibility of treating diabetes with an oral therapy to restore β-cell mass, and highlights a tractable pathway for future drug discovery efforts.

## Abstract (web summary)

All forms of diabetes eventually lead to a reduction in insulin-secreting pancreatic β-cells. Here, the authors report aminopyrazine derivatives, which induce proliferation of rodent as well as human β-cells and improve glucose metabolism in a mouse model of type 1 diabetes.

## Graphical abstract

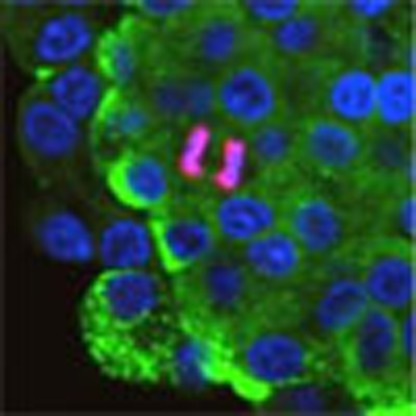

All forms of diabetes mellitus are associated with a decrease in pancreatic β-cell mass. Patients with type 1 diabetes (T1D) have a dramatic reduction in β-cell mass, leading to insulin insufficiency and hyperglycaemia (reviewed in ref. 1). In type 2 diabetes, insulin resistance causes a compensatory expansion of β-cells and increased plasma insulin levels^2^,^3^. However, frank diabetes develops over time as β-cell mass decreases. Notably, a majority of genes identified in genome-wide association studies of type 2 diabetes are regulators of β-cell mass and/or β-cell function^4^. Finally, insufficient β-cell mass and insulin secretion also cause mature onset diabetes of the young and gestational diabetes. Therefore, approaches to increase functional pancreatic β-cell mass may lead to improved therapeutic options for treatment of many forms of diabetes.

β-cell replication maintains functional β-cell mass in adult mice^5^,^6^ and humans^7^, and several studies have shown proliferation in primary β-cells following a variety of genetic or pharmacologic interventions^2^,^8^,^9^,^10^,^11^,^12^,^13^,^14^,^15^,^16^,^17^. While a large number of hormones, small molecules, growth factors and nutrients are capable of inducing primary rodent β-cell replication, only harmine has been demonstrated to stimulate an increase in proliferation of adult primary human β-cells^17^,^18^.

Here we build upon previous work from our group^19^ and describe a new series of compounds, the aminopyrazines, that are capable of stimulating the proliferation of primary rodent and human islets *in vitro* and *in vivo*. Furthermore, we show that these compounds inhibit the nuclear factor of activated T-cells (NFAT) kinases, dual specificity tyrosine-phosphorylation-regulated kinase 1A (DYRK1A) and glycogen synthase kinase-3 beta (GSK3B), leading to NFAT nuclear localization, and that this inhibition is required for β-cell proliferation. These findings may provide an opportunity for therapeutic intervention in diabetes.

## Results

### Aminopyrazines stimulate β-cell proliferation

**Figure 1:**
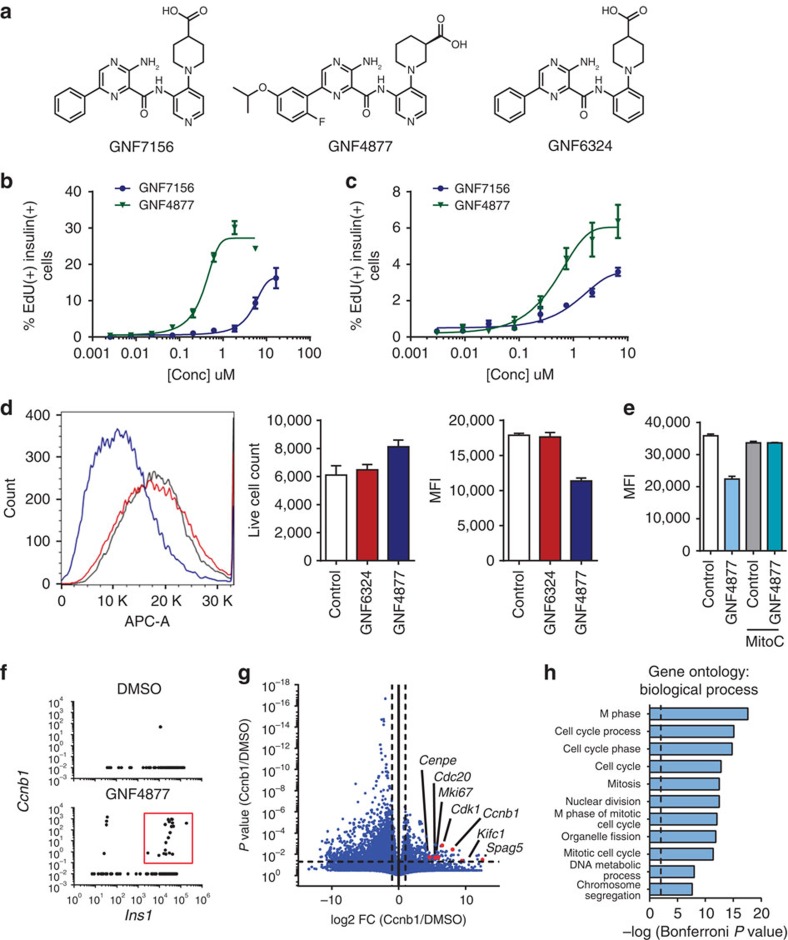
Aminopyrazine compounds induce primary rodent and human β-cell proliferation. (**a**) Structures of the aminopyrazine (AP) compounds GNF7156, GNF4877 and GNF6324. (**b**–**c**) Dose response of GNF7156 and GNF4877-induced EdU incorporation into β cells from rat (**b**) or human (**c**) islets. (**d**–**e**) Cell division induced by GNF4877. (**d**) Rat islets were loaded with eFluor670 and subsequently treated with GNF4877 or the inactive analogue GNF6324 for 5 days. Fluorescence was determined by FACS and represented as mean fluorescence intensity (MFI; mean±s.d.; *n*=5). (**e**) Dilution of eFluor670 dye was abrogated by cell cycle inhibitor Mitomycin C (MitoC). (**f**–**h**) Single cell RNA sequencing from rat islets. (**f**) Single cell RNA sequencing of rat islets reveals a greater number of *Insulin 1* (*Ins1*) and *Ccnb1* co-positive cells with GNF4877 treatment (red box). (**g**) Volcano plot comparing gene expression of *Ccnb1*/ *Ins1* positive cells from GNF4877 treatment (red box) to *Ins1* expressing cells in DMSO reveals a significant increase in expression of cell cycle genes (**g**) and gene ontology biological processes (**h**) strongly associated with cell cycle progression.

We synthesized >300 compounds from an aminopyrazine scaffold that had been identified in a high-throughput screen for β-cell proliferation^19^. Exemplar compounds GNF7156 and GNF4877 (Fig. 1a; Supplementary Methods) stimulate proliferation of reversibly immortalized mouse beta (R7T1) cells, with EC_50_ (half-maximal effective concentration) of 0.66 μM (GNF4877) and 2.2 μM (GNF7156) (Supplementary Fig. 1a). We used incorporation of EdU into primary islet cells as a measure of DNA synthesis in S phase. By this measure, GNF4877 and GNF7156 induced 15–30% of adult rat primary β-cells to enter the cell cycle (Fig. 1b; Supplementary Fig. 1b). Similar results were obtained when cell cycle progression was measured using bromodeoxy-uridine (BrdU) incorporation, Ki67 staining or by using Pdx1 as a marker of β-cells rather than insulin (Supplementary Fig. 1c). Both aminopyrazine compounds caused EdU incorporation into 3–6% of β-cells from dissociated adult primary human islets (Fig. 1c) and similar results were observed using primary mouse islets (Supplementary Fig. 1d). Each compound was tested in at least five human donors with consistent results (EC_50_ GNF7156, 0.76±0.49 μM and GNF4877, 0.54±0.171 μM).

We determined whether treatment of primary islet cells with aminopyrazine compounds caused β-cell division by measuring dilution of the florescent vital dye (eFluor670). Rat islet cells loaded with eFluor670 and subsequently treated with GNF4877 for 5 days had a decreased intensity of eFluor670 relative to control–treated cells, confirming that aminopyrazine treatment induces bona fide cell division in these cells (Fig. 1d). This decrease in staining of eFluor670 was dependent on cellular proliferation, as it did not occur in the presence of mitomycin C, a cell cycle inhibitor (Fig. 1e). GNF6324, a closely related analogue of GNF4877 did not induce EdU incorporation in rat β-cells, nor cause a decrease in eFluor670 staining in rat islet cells (Fig. 1d). The extent of proliferation of human islet cells was too low to be detected with this method, consistent with the lower level of EdU incorporation induced in human islets. Microscopic examination of primary adult human β-cells revealed cells in the process of division in GNF4877-treated islets, but not in vehicle-treated control islets (Supplementary Fig. 1c,e).

To further evaluate the effects of GNF4877 on cell cycle control, we performed global transcriptional analysis. Due to the limited number of proliferating cells among the total islet cell population, single cell RNA sequencing was utilized to evaluate the transcriptional profile of individual cells from primary rat islets. Consistent with GNF4877 eliciting β-cell proliferation, we observed an increase in the number of β-cells co-expressing *Insulin 1* and genes involved in the cell cycle including the M phase marker *Cyclin B1* (Fig. 1f). Comparison of *Cyclin B1*-expressing cells from GNF4877-treated islets to β-cells from dimethylsulfoxide (DMSO)-treated islets further revealed a significant increase in expression of genes associated with full cell cycle progression, including cytokinesis, and enrichment of Gene Ontology categories for proliferation (Fig. 1g,h).

Since some known β-cell mitogens have been shown to increase the DNA damage response, we asked whether EdU incorporation into human or rat β-cells might be reflecting DNA damage and repair rather than entry into S phase. DNA damage-inducing agents phleomycin, methyl-methanesulfonate or streptozotocin (STZ) led to increased terminal deoxynucleotidyl transferase dUTP nick end labeling (TUNEL), p53 binding protein (53bp1) and phospho-γH2aX, but not EdU incorporation (Supplementary Figs 2,4 and 5). Overexpression of CDK6 and Cyclin D1 increased TUNEL/EdU double-positive cells (Supplementary Fig. 2a–c) in agreement with previous reports^20^. Aminopyrazine compounds increased EdU incorporation without affecting the average number of TUNEL-positive β-cells (Supplementary Fig. 2a–e). Notably, in rat β-cells that are proliferating in the absence of aminopyrazine compound, increased nuclear 53BP1 puntae are observed in EdU-positive cells, suggesting the DNA damage response is a normal component of β-cell replication (Supplementary Fig. 4a). This observation is consistent with increased DNA damage markers during cell cycle in other cells types and the known role of these proteins in maintaining genomic stability in non-homologous end-joining and homologous recombination^21^,^22^. We conclude that EdU incorporation in response to aminopyrazine compounds is not reflecting induction of double-strand DNA breaks and is instead likely to reflect entry into S phase.

### GNF4877 expands human islet mass with retention of function

**Figure 2:**
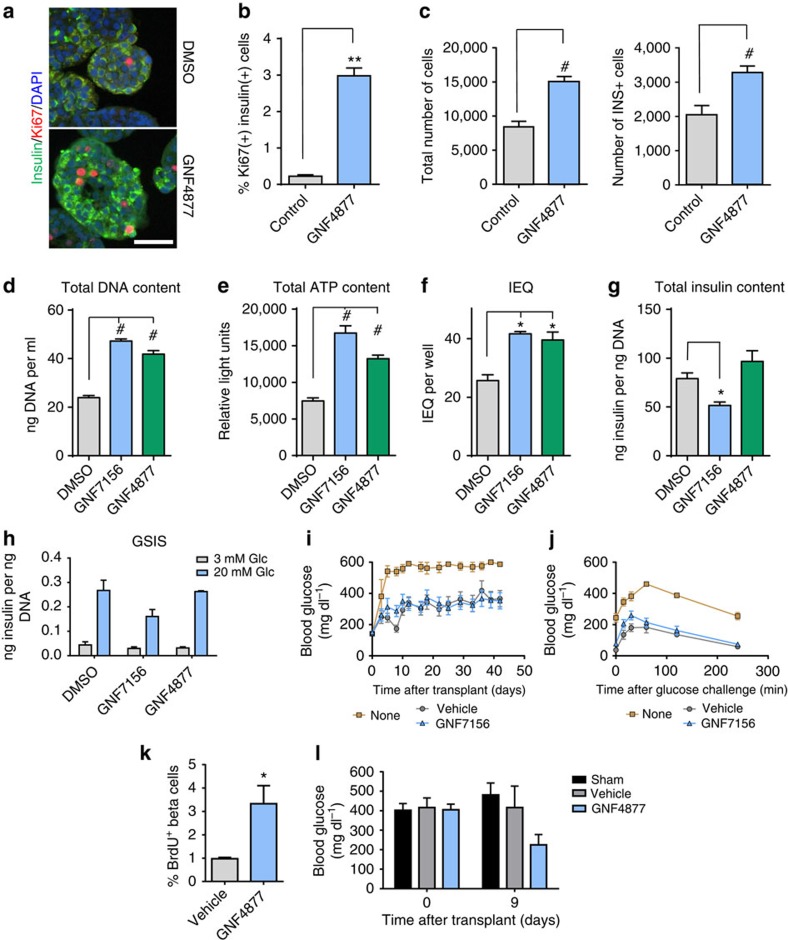
AP compounds expand human islets *ex vivo* with retention of function after transplantation. (**a**–**c**) Treatment of intact primary human islets with GNF4877 for 8 days results in increased beta cell numbers relative to vehicle control–treated islets. (**a**) Immunofluorescence for insulin, Ki67 and DAPI on DMSO or GNF4877-treated human intact islets (scale bar, 50 μm). (**b**) Quantification of Ki67+ as a percent of total insulin+ cells (*n*=3). (**c**) Total number of islet cells and total number of insulin+ cells after 8 days of treatment of human islets with GNF4877 or vehicle (*n*=24 islets; bars represent mean±s.d.; **P<0.01, ^#^*P*<0.0001, Student's *t*-test. (**d**–**j**) Primary human islets were treated for 7 days with the AP compounds or vehicle control (6.7 μM GNF7156 or 2 μM GNF4877; *n*=9 per condition) and then tested for (**d**) total DNA content, (**e**) total ATP content, (**f**) islet equivalent units (IEQ), (**g**) total insulin content and (**h**) glucose stimulated insulin release (GSIS) (*P<0.05, ^#^*P*<0.0001, Student's *t*-test). (**i**–**j**) GNF7156 and DMSO-expanded human islets are functionally equivalent when equal numbers were transplanted into the kidney capsule of STZ-induced diabetic NOD–SCID mice: (**i**) Fed blood glucose and (**j**) oral glucose tolerance test. Data are shown as mean±s.d. (six mice per group; transplanted with islets from a single human donor). (**k**–**l**) NSG mice were transplanted with a sub-optimal dose of human islets under the kidney capsule before treatment with GNF4877 (oral, 50 mg kg^−1^, twice daily) or vehicle control. GNF4877-treated NSG mice transplanted with human islets demonstrated increased BrdU incorporation into insulin-positive cells by immunohistochemistry (**k**) and display improved glucose control as measured by fed glucose levels (**l**). Data shown as mean±s.d.; (*n*=6 mice/group, *P<0.05, Student's *t*-test).

Given that aminopyrazine compounds cause proliferation of human and rat β-cells, we asked if this was accompanied by expansion of β-cells *in vitro*. GNF4877 promoted a 20-fold increase in the number of Ki67-positive insulin-positive cells in intact human islets (Fig. 2a,b). GNF4877 treatment of human islet cells for 7–8 days led to greater numbers of both total islet and β-cells relative to control–treated cultures (Fig. 2c; Supplementary Fig. 3a–e). In most donors, there was an absolute decrease in cell numbers during the time of culture, even in the presence of GNF4877 (Supplementary Fig. 3b,e). However, in one donor where cell loss was minimal in the control culture, we observed an increase in total cell and β-cell number after treatment with GNF4877 (Supplementary Fig. 3c,d), consistent with expansion through proliferation. Together, these data support a role for β-cell proliferation induced by GNF4877, but do not rule out an additional effect of enhancing β-cell survival.

We tested whether aminopyrazine treatment of islets compromised their ability to secrete insulin *in vitro* and, after transplantation, *in vivo*. Human islets treated for 7 days with GNF7156 or GNF4877 *in vitro* showed increases in DNA and ATP content and an increase in islet equivalent units (IEQ) compared with vehicle-treated cultures (Fig. 2d–g, representative results from three human donors). Although GNF7156 treatment slightly reduced insulin content, both GNF7156- and GNF4877-treated islets maintained insulin secretory capacity (Fig. 2g,h). In addition, GNF7156 and vehicle-treated islets maintained the ability to preserve euglycemia *in vivo* after transplantation into STZ-treated NOD.CB17-Prkdc^scid^/J (NOD–SCID) mice (Fig. 2i,j). Similar results were observed when human islets were treated with GNF4877 before transplantation.

We asked if exposure of adult human β-cells to GNF4877 *in vivo* would stimulate β-cell proliferation. We transplanted a sub-optimal dose of human islets under the kidney capsule of immune-compromised (NOD.Cg-*Prkdc*^*scid*^ *Il2rg*^*tm1Wjl*^/SzJ; NSG), STZ-treated mice and dosed the recipient mice with vehicle or GNF4877 for 9 days. Indeed, compared with vehicle-treated animals, GNF4877-treated animals showed greater BrdU incorporation into insulin-positive cells in the graft (Fig. 2k). The GNF4877-treated mice showed a trend towards improved glucose control demonstrating functionality of the transplanted islets (Fig. 2l). Taken together, these findings demonstrate that aminopyrazine compounds lead to proliferation of functional primary human β-cells.

### GNF4877 improves glycaemic control in a mouse model of T1D

**Figure 3:**
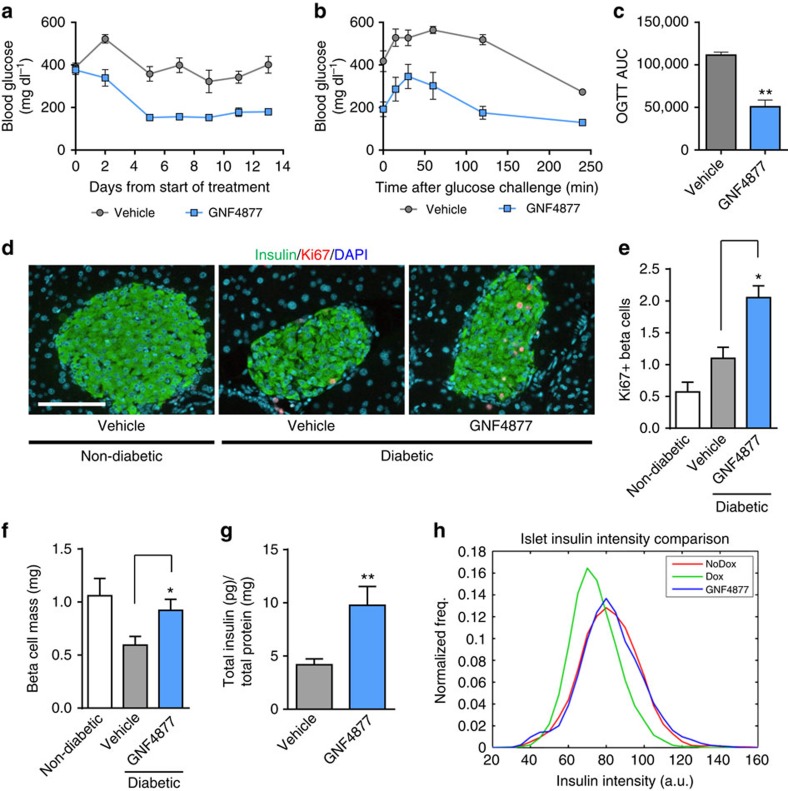
AP compounds improve glucose control and β-cell mass in the RIP-DTA diabetes model. (**a**–**h**) Diabetic RIP-DTA mice were treated with GNF4877 (oral, 50 mg kg^−1^ twice daily) after disease induction (when blood glucose reached 400 mg dl^−1^). (**a**) GNF4877 caused progressive reduction of hyperglycemia (fed glucose measurement) relative to vehicle-treated animals. (**b**–**c**) GNF4877-treated animals showed improved oral glucose tolerance following 14 days of dosing. (**c**) Area under the curve (AUC) from oral glucose tolerance test (OGTT) data was significantly improved by GNF4877 treatment (*n*=8 mice/group; data shown as mean±s.d.; *P<0.05; **P<0.01, Student's *t*-test). (**d**–**h**) Histological analysis for insulin and Ki67 from pancreata of non-diabetic and diabetic mice after 14 days of treatment with GNF4877 for 14 days reveals increased percentage of Ki67+ Ins+ cells (**d**,**e**), increased β-cell mass (**f**), increased insulin content (**g**), and mean fluorescent intensity of insulin (**h**) compared with vehicle-treated control mice. (*n*=8 mice/group; data shown as mean±s.d.; *P<0.05; **P<0.01, Student's *t*-test). Scale bar, 100 μm.

We evaluated the effects of GNF4877 in a mouse model of T1D, in which inducible expression of diphtheria toxin A in β-cells leads to their ablation and consequent diabetes Tg(Ins2-rtTA)2Efr Tg(teto-DTA)1Gfi/J; (RIP-DTA) mice; ref. 23). Treatment with GNF4877 was initiated at the onset of overt diabetes (blood glucose >400 mg dl^−1^) and caused a progressive reduction of non-fasting blood glucose levels (Fig. 3a). GNF4877-treated mice showed dramatically improved glucose tolerance compared with vehicle-treated control mice (Fig. 3b,c and Supplementary Fig. 7a). GNF4877 treatment was well tolerated as mice did not show any difference in body weight (Supplementary Fig. 7b), appearance or behaviour compared with vehicle-treated mice. Morphometric analysis of pancreas sections showed increased insulin staining (Supplementary Fig. 6a), proliferating β-cells, β-cell mass (∼1.5 fold) and insulin content (∼twofold) in GNF4877-treated compared with vehicle-treated diabetic RIP-DTA mice (Fig. 3d–h). Consistent with these observations, we observed an increase in insulin intensity per islet in GNF4877-treated animals compared with vehicle-treated diabetic animals (Fig. 3h; Supplementary Fig. 6b). While intra-islet Ki67^+^ cells were increased, there was no increase in extra-islet proliferation in the pancreas from GNF4877-treated animals (Supplementary Fig. 6c). GNF4877-treated diabetic RIP-DTA mice showed improved oral glucose tolerance without a significant difference in insulin tolerance suggesting improved glycaemic control was not due to increased insulin sensitivity (Supplementary Fig. 7a,c). These data demonstrate a significant increase in β-cell mass in diabetic mice treated with GNF4877, which is associated with, and likely causative for, improved glycaemic control.

### β-Cell proliferation through inhibition of Gsk3b and Dyrk1a

**Figure 4:**
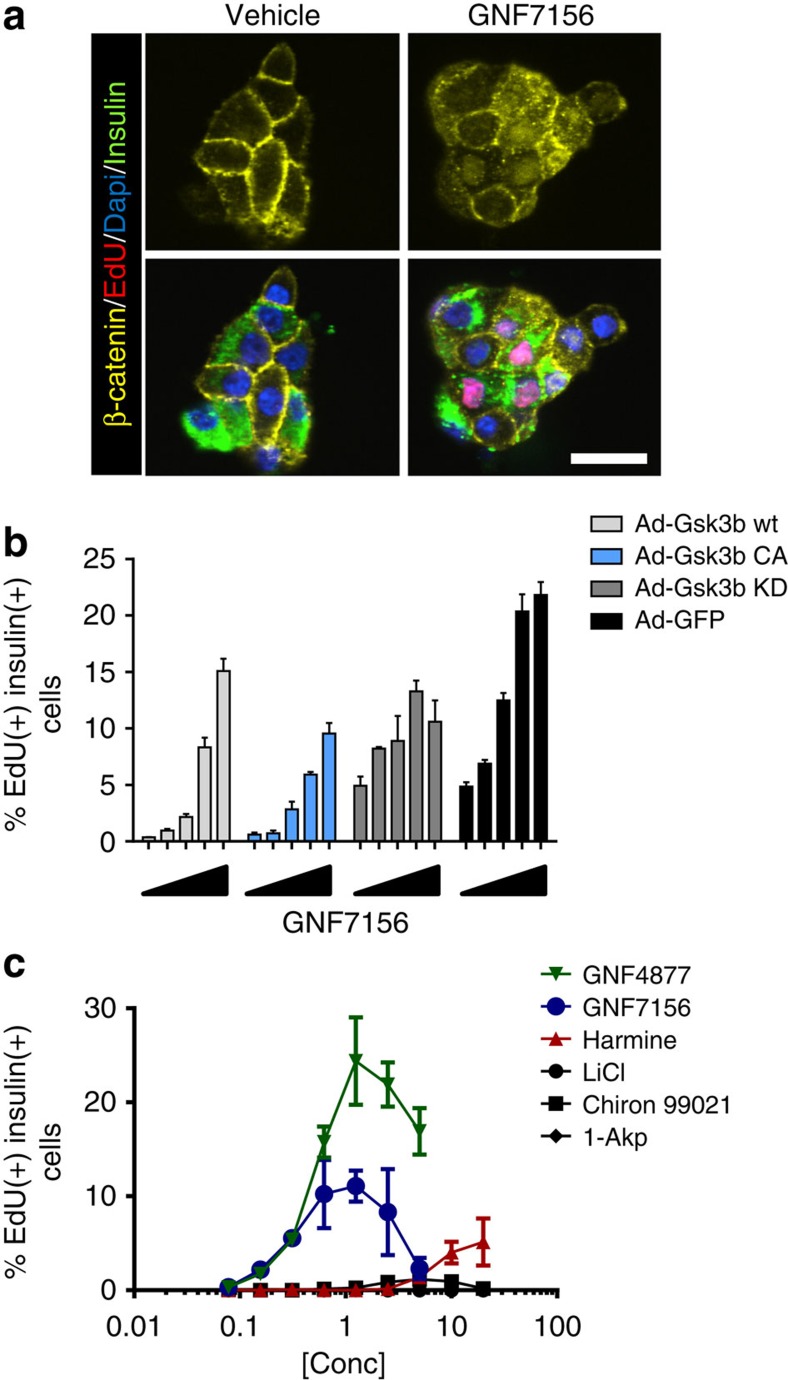
Aminopyrazines inhibit Gsk3b. (**a**) GNF7156 induces nuclear localization of β-catenin in EdU and insulin co-positive cells from primary rat islets. (**b**) Adenoviral overexpression of wild type (wt), constitutively active (CA) and kinase-dead (KD) Gsk3b partially inhibited GNF7156-induced β-cell proliferation in primary dispersed rat islets as judged by EdU incorporation (*n*=8/group; mean±s.d.). (**c**) Gsk3b inhibitors LiCl, 1-Akp and Chiron99021 induce minimal or no proliferation of rat β-cells from dispersed islets, whereas the Dyrk1a inhibitor harmine induces EdU incorporation into insulin-positive cells (*n*=3 per data point; mean±s.d.). Scale bar, 20 μm.

Related aminopyrazine compounds have been reported as selective Gsk3b inhibitors^24^, and Gsk3b has been implicated in rodent β-cell development and proliferation^25^,^26^,^27^,^28^. Therefore, we evaluated a potential role for Gsk3b inhibition in the mechanism of action for aminopyrazine-induced β-cell proliferation. GNF7156 and GNF4877 potently inhibit Gsk3b in a biochemical kinase assay, (IC_50_ (half-maximal inhibitory concentration) 40 and 16 nM, respectively) and induce a Wnt-driven luciferase reporter gene (Supplementary Fig. 8a). GNF7156 induced β-catenin nuclear translocation (Fig. 4a), consistent with cellular Gsk3b inhibition. Induction of rat β-cell proliferation by GNF7156 was blunted by ectopic expression of wild-type or constitutively active Gsk3b (ref. 29), but less by kinase-dead Gsk3b (ref. 30; Fig. 4b). Gsk3b inhibitors from other scaffolds (for example, 1-Akp, Chiron99021, LiCl; ref. 31) also stimulated proliferation of rat β-cells, although to a significantly lower extent than GNF7156 and GNF4877 (Fig. 4c) and without any significant effects on human β-cell proliferation (Supplementary Fig. 8c). Overexpression of Gsk3b only partially inhibited aminopyrazine-induced β-cell proliferation (Fig. 4b), and β-cells can proliferate and function normally in the absence of β catenin^32^. Together, these data led us to conclude that Gsk3b inhibition alone could not explain the effect of aminopyrazine compounds on β-cell proliferation, and prompted a search for other targets.

**Figure 5:**
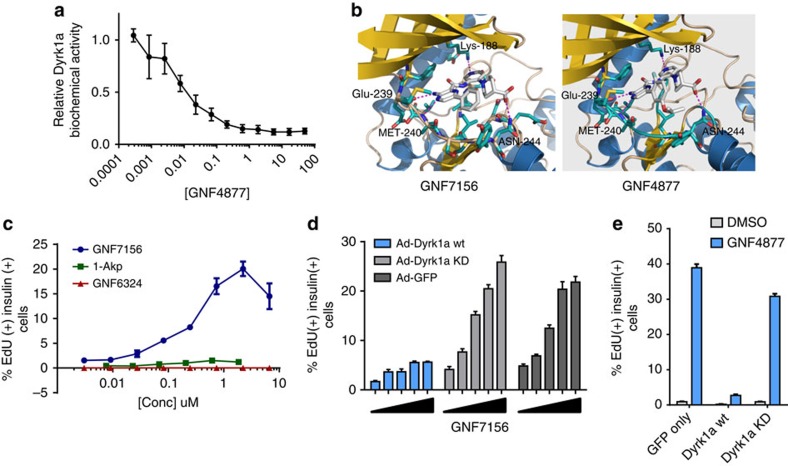
Dyrk1a inhibition is a critical mechanism of AP-stimulated β-cell proliferation. (**a**) GNF4877 potently inhibits Dyrk1a kinase activity in a biochemical assay (data point is mean±s.d., *n*=3). (**b**) Molecular modelling of GNF7156 and GNF4877 in the Dyrk1a crystal structure. (**c**) GNF6324, a Gsk3b inhibitor compound closely related to GNF7156, but lacking Dyrk1a activity, does not induce proliferation of primary rat β-cells. Dispersed primary rat islets were treated with the indicated compounds and β-cell proliferation was measured by EdU incorporation into insulin-positive cells (*n*=3 per data point; mean±s.d.). (**d**–**e**) Adenoviral overexpression of wild type (wt) but not kinase-dead (KD) Dyrk1a blocked GNF7156 (**d**) or GNF4877 (**e**) induced β-cell proliferation in dispersed rat islets. Proliferation of β-cells was measured by EdU incorporation into insulin-positive cells (*n*=3; mean±s.d.).

From human kinome scanning (Ambit KINOMEscan), we noted that GNF7156 and GNF4877 potently inhibited the dual-specificity tyrosine-(Y)-phosphorylation-regulated kinase 1A (Dyrk1a, IC_50_ 100 and 6 nM, respectively; Fig. 5a; Supplementary Table 1). We focused on Dyrk1a because other kinases inhibited by aminopyrazine compounds were not highly expressed in β-cells and because Dyrk1a was known to inhibit the NFAT pathway that was previously implicated in β-cell proliferation^33^,^34^,^35^. We used molecular modelling to dock the aminopyrazine compounds into the published structure of Dyrk1a (protein databank accession code 2vx3.pdb; Fig. 5b). Using this structural information, we synthesized a closely related compound, GNF6324 (Fig. 1a; Supplementary Methods), that lacked Dyrk1a inhibitory activity (IC_50_>50 μM), but retained similar potency to GNF7156 on Gsk3b. Consistent with a critical role for Dyrk1a inhibition, and confirming that Gsk3b inhibition is insufficient to drive full β-cell proliferation, GNF6324 was completely inactive in both rat and human β-cell proliferation assays (Figs 1d and 5c, Supplementary Fig. 8c), but capable of inducing nuclear localization of β-catenin (Supplementary Fig. 8b).

β-cell proliferation induced by GNF7156 or GNF4877 was inhibited by overexpression of wild-type Dyrk1a, but not by kinase-dead Dyrk1a (ref. 36), supporting a role for the kinase activity of Dyrk1a in aminopyrazine-induced β-cell proliferation (Fig. 5d,e). Notably, the effect of overexpression of wild-type Dyrk1a (Fig. 5d) was more pronounced than that of overexpression of wild-type Gsk3b (Fig. 4b). Structurally distinct inhibitors of Dyrk1a (including 5-IT, harmine and TG003 (ref. 37)) induced β-cell proliferation in rat and human dispersed islet assays (Fig. 4c; Supplementary Fig. 8c–f). These data support a conclusion that Dyrk1a kinase inhibition is sufficient to induce β-cell proliferation.

Finally, we asked whether dual inhibition of Dyrk1a and Gsk3b would be more effective in inducing proliferation of β-cells than inhibition of either alone. Indeed, the combination of the highly selective Gsk3b inhibitor Chiron99021 with a selective Dyrk1a inhibitor TG003 resulted in additive induction of primary rat β-cell proliferation (Supplementary Fig. 8f). These pharmacologic and genetic data further validated Dyrk1a and Gsk3b as critical regulators of β-cell proliferation.

### Aminopyrazine compounds regulate NFAT nuclear localization

**Figure 6:**
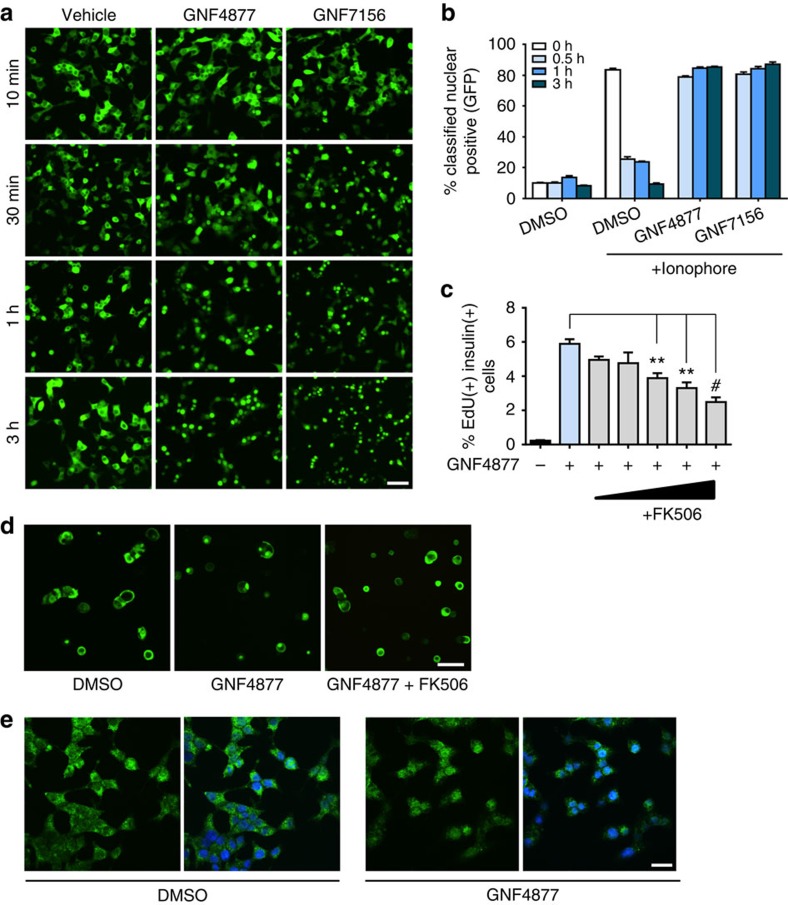
AP compounds induce β-cell proliferation through activation of the calcineurin–NFAT pathway. (**a**) AP compounds stimulate NFATc1-GFP nuclear localization. Two days after infection of Ins1 cells with adenoviral NFATc1-GFP, cells were treated with GNF4877, GNF7156 or vehicle control for the indicated time before high content imaging for NFATc1-GFP cellular localization. Scale bar, 50 μm. (**b**) AP compounds inhibit NFATc1 nuclear export after induction of NFATc1 nuclear localization with ionophore. Following ionophore withdrawal, cells were treated with GNF4877, GNF7156 or DMSO for the indicated times and NFATc1-GFP localization was monitored by high content imaging. (**c**) Primary human β-cell proliferation induced by GNF4877 (2 μM) was inhibited by FK506 (12 nM to 1 μM) in the *ex vivo* human dissociated islet assay (*n*=3; mean±s.d.; **P<0.001, ^#^*P*<0.0001, ANOVA). (**d**–**e**) Endogenous NFATc1 and NFATc3 are nuclear localized by treatment with GNF4877. Primary rat islets were treated with GNF4877 or DMSO control before immunofluorescent staining for endogenous NFATc1 (**d**) or NFATc3 (**e**). Scale bar, 50 μm.

Both Dyrk1a and Gsk3b are negative regulators of the NFAT pathway^33^,^38^,^39^, and this pathway is fundamentally important for murine β-cell proliferation^33^,^34^,^35^. At low glucose, NFATc1-GFP was largely cytosolic in primary β-cells. GNF7156 and GNF4877 caused marked nuclear localization of NFATc1-GFP (Fig. 6a). To determine if aminopyrazine compounds inhibited NFATc1 nuclear export, we treated cells with a calcium ionophore to induce NFATc1 nuclear localization; then followed NFATc1-GFP localization after withdrawal of ionophore. In vehicle-treated cells, nuclear NFATc1 levels returned to baseline levels after 3 hours. By contrast, aminopyrazine-treated cells retained nuclear NFATc1 (Fig. 6b), demonstrating inhibition of NFATc1 nuclear export. Human β-cell proliferation induced by GNF4877 was reduced by co-treatment with FK506 (Fig. 6c) or CsA consistent with a role for calcineurin in this process. FK506 partially blocked the GNF4877-stimulated nuclear localization of NFATc1 (Fig. 6d; Supplementary Fig. 9c). Together, these results point to the Dyrk1a-NFAT pathway as the key mediator for aminopyrazine-induced β-cell proliferation.

**Figure 7:**
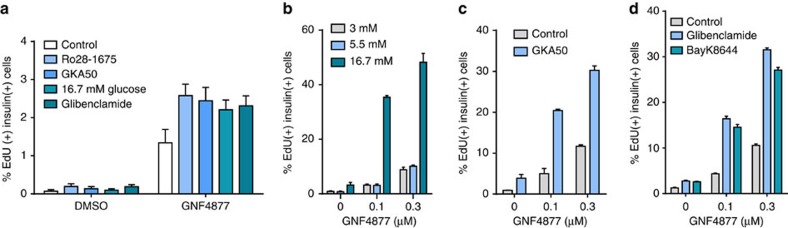
Calcium signaling regulates AP-induced β-cell proliferation. (**a**–**d**) Elevation of intracellular Ca^2+^ levels in β cells augments the response to AP compounds. (**a**) Primary human islets were treated with glucokinase activators (Ro28-1675 or GKA50), high glucose (16.7 mM glucose) or the sulfonylurea (Glibenclamide)±a sub-optimal concentration (0.1 μM) of GNF4877. β-cell proliferation was measured by EdU incorporation into insulin-positive cells (*n*=3 per data point; mean±s.d.). (**b**–**d**) Combination of GNF4877 with agents that elevate intracellular calcium increases proliferation of rat β-cells. Concentrations of GNF4877, below the EC_50_ for rat or human β-cell proliferation, are effective in inducing proliferation in the presence of agents that elevate intracellular calcium: (**b**) high glucose; (**c**) the glucokinase activator GKA50 or (**d**) glibenclamide (a sulfonylurea receptor 1 inhibitor) or Bay K8644 (an L-type Ca^2+^ channel activator).

Homeostatic β-cell replication is systemically controlled by glucose metabolism^14^. High glucose concentrations and glucokinase activators (GKAs) increase Ca^2+^ signalling in β-cells, and increased intracellular Ca^2+^ leads to activation of calcineurin and nuclear translocation of NFATc proteins^40^. We reasoned that agents that increased intracellular Ca^2+^ would activate calcineurin, leading to dephosphoylation and activation of NFAT. Such agents might then further drive β-cell replication in the context of aminopyrazine inhibition of Gsk3b and Dyrk1a. Indeed, concentrations of GNF4877 well below the EC_50_ for β-cell proliferation were able to induce proliferation in the presence of high glucose or pharmacological activators of glucokinase (Fig. 7a–c; Supplementary Fig. 10d). Finally, increasing intracellular Ca2^+^ with glibenclamide (a sulfonylurea receptor 1 inhibitor) or Bay K8644 (an L-type Ca2^+^ channel activator) showed additive activity with GNF4877 (Figs 7a,d). Similar results were observed when GNF7156 was used (Supplementary Fig. 10a–c). These experiments provide further support to the hypothesis that aminopyrazine compounds stimulate β-cell proliferation through a Dyrk1a-mediated pathway.

## Discussion

**Figure 8:**
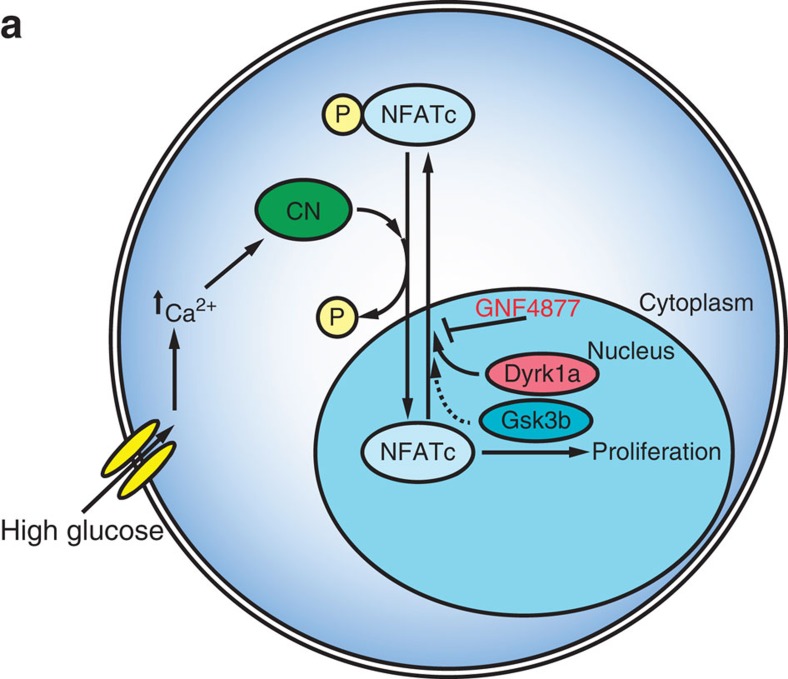
Model of AP-stimulated β-cell proliferation. (**a**) In the presense of high glucose, beta cells increase intracellular Ca^2+^ levels leading to activation of calcineurin (CN). CN dephosphorylates NFATc proteins such as NFATc1 leading to nuclear import. Previous studies demonstrated a critical role for the CN–NFATc pathway in the regulation of β-cell proliferation. Nuclear NFATc kinases Dyrk1a and Gsk3b phosphorylate NFATc proteins leading to nuclear export. AP compounds, such as GNF4877, inhibit Dyrk1a and Gsk3b leading to blockade of NFATc nuclear export and increased β-cell proliferation.

Aminopyrazine treatment of rat and human primary β-cells induces entry into cell cycle when measured by staining for Ki67, EdU and BrdU, and through unbiased gene expression profiling of single β-cells. We further show, using dilution of a vital dye that aminopyrazine treatment causes bona fide cell division of rat islet cells and, using microscopy, that aminopyrazine treatment causes division of human β-cells. Through interrogation of compound mechanism of action, we discovered that the aminopyrazine compounds' activity on β-cell proliferation critically relies on inhibition of DYRK1A and GSK3B (Fig. 8). Notably, aminopyrazine treatment does not compromise β-cell function *in vitro* or *in vivo.* Nor does treatment with aminopyrazine compounds cause an increase in a DNA damage response beyond that associated with spontaneous proliferation. Thus, aminopyrazine compounds have the potential to produce a functional expansion of β-cells.

Our data are consistent with a recent study that identified harmine as a DYRK1A inhibitor that induces β-cell proliferation^17^. The use of DYRK1A inhibitors from three different scaffolds (aminopyrazine, harmine and 5-IT) support the concept that DYRK1A inhibition is sufficient to drive β-cell proliferation. However, our findings demonstrate that dual inhibition of GSK3B and DYRK1A more robustly stimulates β-cell proliferation, even when compared directly with harmine or 5-IT (Fig. 4c; Supplementary Fig. 8f). The aminopyrazine compounds are not exclusive DYRK1A/GSK3B inhibitors and thus we cannot completely rule out involvement of other potential targets in the effects of these compounds.

Our model is consistent with prior data on the role of NFAT in mouse β-cell proliferation. Inhibition of the NFAT pathway through genetic deletion of calcineurin in β-cells caused reduced β-cell mass through impaired proliferation^34^,^35^. Similarly, pharmacological inhibition of calcineurin by FK506 or cyclosporin A in both humans and rodents leads to decreased pancreatic and plasma insulin. Indeed, 15–30% of patients taking FK506 or cyclosporine A develop diabetes^35^,^41^. Conversely, β-cell-specific expression of active NFATc1 rescues these phenotypes in β-cell-specific mouse knockouts of calcineurin^34^,^35^. We propose that pharmacological inhibition of DYRK1A and GSK3B can drive supra-physiological levels of NFAT activation and an increase in β-cell proliferation beyond that achievable even under conditions of increased glucose load^14^. We further note that additional substrates (other than NFAT) for DYRK1A identified in other cell types might contribute to the role of this kinase in β-cell regulation such as stabilization of Cyclin D1 (ref. 42) or assembly of the DREAM complex^43^.

Heterozygous *Dyrk1a* knockouts have impaired β-cell development and a decrease in β-cell mass^44^, a phenotype that seems inconsistent with the data presented here. Due to the specific effects of aminopyrazine compounds on adult islets, our data suggest that Dyrk1a plays a different role in islet development through embryogenesis compared with homeostasis in the adult organism.

Patients with Down syndrome (trisomy 21) have increased gene dosage of genes in the Down syndrome critical region (DSCR), which includes DYRK1A. Patients with Down syndrome have a greater than fourfold increase in the prevalence of type 1 diabetes^45^,^46^. Genetic studies demonstrate increased gene dosage of Dyrk1a and another gene in the DSCR (DSCR1) combine to reduce NFAT signalling leading to Down syndrome-like features in mice^33^. We speculate that increased DYRK1A gene dosage (as observed in Down syndrome patients) results in reduced β-cell proliferation and an increased risk of diabetes.

The utility of aminopyrazine compounds in treating diabetes can only be assessed after more extensive toxicity testing. However, we note that dosing of aminopyrazine compounds at pharmacologically active levels for 21 days to mice was not associated with weight loss, gross changes in animal appearance or behaviour. Dyrk1a is widely expressed and effects of inhibition beyond the β-cells can be expected. For example, recent reports of mutations in DYRK1A in autism^47^,^48^ highlight possible risks of inhibiting this kinase, though limiting brain exposure of an inhibitor during development and early childhood might help ameliorate the risk.

In summary, we describe small molecules that potently induce adult primary human β-cell proliferation *in vitro* and *in vivo*, and that improve glucose control in a mouse model of diabetes after oral dosing. These compounds may provide a path forward to develop new drugs to treat diabetes.

## Methods

### Statistical analysis

For all experiments, two-sided *t*-tests were used to evaluate differences between two groups and analysis of variance (ANOVA) was used for multiple groups. To perform statistical analysis, at least three independent experiments from three different organ donors were performed for human islets; at least three independent experiments were performed for rat islets and pancreatic β-cell lines, as reported in figure legends. Data are presented as mean±s.d. For all *in vivo* animal studies, eight mice per group is required to reliably detect changes in glycemic control and β-cell mass based on historical data to achieve statistical significance level of *α*=0.05.

### Animal experiments

All animal care and experimental procedures were approved by the Institutional Animal Care and Use Committee (IACUC) of Genomics Institute of the Novartis Research Foundation and strictly followed the NIH guidelines for humane treatment of animals. The double transgenic RIP-DTA male mice (Tg(Ins2-rtTA)2Efr Tg(teto-DTA)1Gfi/J; Jackson Laboratory, Stock # 008755) were bred in-house and were maintained in a 12-h light/12-dark cycle environment with controlled temperature, humidity and ventilation. Animals were housed 3–5 mice per cage and had free access to water and standard laboratory chow. To induce pancreatic β-cell ablation and hyperglycaemia, doxycycline (200 μg ml^−1^, Sigma-Aldrich) was provided in the drinking water for 5 days. Blood glucose levels were measured from tail-vein blood with a glucometer (AlphaTrak2 Monitoring System; Abbott). Experimental groups of 14 male mice each were randomized based on similar body weights (28.8±2.4 g), age (82±2 days), and blood glucose levels (428±96 mg dl^−1^). Group sizes of eight animals each are sufficient to reliably detect changes in glycaemic control and β-cell mass based on historical data with reference compounds; an additional six mice were added to each group to assess total pancreatic insulin levels. Doxycycline-induced diabetic mice were treated with either vehicle (0.5% MC/0.5% Tween-80), GNF7156 (50 mg kg^−1^ formulated in 0.5% MC/0.5% Tween-80, once a day) or GNF4877 (50 mg kg^−1^ formulated in 0.5% MC/0.5% Tween-80, twice a day). A sub-set of animals were not given doxycycline treatment and were used as a non-diabetic vehicle–control group. All compounds were administered daily via oral gavage (4 ml kg^−1^). Body weight and blood glucose were monitored every third day. An oral glucose tolerance test was performed after an overnight fast on day 12. For the insulin tolerance test, mice were fasted for 4 h before the administration of insulin (Novolin, Novo Nordisk, 0.7 U kg^−1^ body weight, i.p.). Pancreatic insulin content was determined by MSD (meso-scale discovery). Insulin ELISA (enzyme-linked immunosorbent assay) kit from whole pancreas lysate from mice (*n*=6) treated for 15 days with vehicle or GNF4877 (50 mg kg^−1^, twice daily). To determine β-cell proliferation and β-cell mass by morphological analyses, pancreata from the rest of animals (*n*=8) were rapidly dissected, fixed in formalin, sectioned and immunostained for insulin, Ki67 and BrdU on day 16. Male NSG mice, aged 8 weeks, were obtained from the Jackson Laboratory (Strain NOD.Cg-Prkdc^scid^ Il2rg^tm1Wjl^/SzJ; Stock number 005557) and housed according to IACUC guidelines. Baseline body weight and tail-vein blood glucose measurements were recorded before diabetic induction. To obtain a diabetic state, animals were given an intraperitoneal dose of STZ (135 mg kg^−1^) and blood glucose was monitored before transplant. Mice were grouped according to body weight and blood glucose level on the day of transplants (72 h after STZ treatment).

### Transplantation experiments

Human islets (obtained commercially from Prodo Laboratories Inc.) were recovered for 48 h before transplantation. On the day of transplantation, mice were anaesthetized with a single i.p. injection of ketamine/xylazine (ketamine 75 mg kg^−1^; xylazine 10 mg kg^−1^). While mice were prepared, islets were counted according to IEQs and prepared into islet suspension of 800 IEQ/mouse in cold PBS. Islets were inserted into the renal pocket and the renal capsule was closed and the kidney was placed back into the abdominal cavity. The incision was closed and mice were given buprenorphrine and flunixin analgesics via subcutaneous injection. Sham kidneys received an equivalent volume of saline injected into the renal capsule. Mice recovered from surgery for 4 days following the transplant and body weight was monitored regularly. After 4 days, mice were given an oral dose of 0.5% MC/Tween vehicle or GNF4877 (50 mg kg^−1^; twice a day). In addition, mice were given an i.p. dose of BrdU (Life Technologies Catalogue number 00-0103; aqueous solution at 0.01 ml g^−1^ according to manufacturer instructions) once daily.

### Histology

Pancreas specimens were harvested, weighed, fixed in 10% neutral buffered formalin and processed using standard paraffin processing techniques. Five micrometre serial-step sections were mounted onto Superfrost Plus slides and air-dried overnight. Samples were coded before histologic analyses resulting in blinded processing and staining. Routine hematoxylin and eosin (H&E) stains were performed on all specimens. Immunostaining was performed using the Ventana Discovery XT system. All epitope retrieval was done on the instrument using CC1 reagent. Chromogenic insulin was detected using guinea pig anti-insulin (Dako Corp A0564) at 1:3,200 and the Blue Map kit, after serum blocking and endogenous biotin blocking was performed, slides were then counterstained with Nuclear Fast Red. Fluorescent insulin and Ki67 double stains used a cocktail of the Dako anti-insulin at 1:1,600 and rabbit monoclonal Ki67 (Thermo RM9106) at 1:100, and were detected using a cocktail of Alexa Fluor 488 and 594-labelled secondary antibodies. Auto-fluorescence was quenched in red blood cells and reduced in connective tissue by treatment with 10 mM copper sulfate. Slides were counterstained with DAPI. 21 slides were collected per block. All slides were imaged on the Hamamatsu Nanozoomer and evaluated for β-cell mass and proliferation.

### Automated image analysis

Automated β-cell mass calculation using MATLAB software: tissue masks were created using chromogenic insulin slides. Co-localization of islets in chromogenic and fluorescent insulin slides was identified by overlaying these two stains to verify the detection of islet regions of interest. Islet regions of interests were then mapped to chromogenic insulin slides to search for β-cell area by chromagen staining. Total tissue area was calculated from tissue mask area, whereas β-cell area is calculated from chromogenic insulin stained area for each section. β-Cell mass was calculated by multiplying the islet/pancreas area ratio by pancreas weight.

### Automated evaluation of intra-islet versus extra-islet proliferation

Insulin/Ki67 stained slides were generated as described above. Rulesets were written to detect and exclude stain artifacts, tissue folds and lymph nodes from pancreas sections. Approximately 500,000 nuclei were counted per pancreas section and binned into either ‘intra-islet' region (defined by the fluorescent insulin stain) or ‘extra-islet' region (remaining tissue). Nuclei with Ki67 stain intensity higher than a pre-defined threshold were classified as Ki67 positive. In each region, intra- or extra-islet, numbers of Ki67-positive nuclei was then divided by total number of nuclei per region. After blinded, automated analysis was complete, images were decoded.

### Cell culture and islet isolation

R7T1 β-cells were obtained from Dr S. Efrat (Tel Aviv University)^49^ and expanded in growth medium (DMEM with 15% horse serum and 2.5% fetal bovine serum (FBS)) in the presence of 10 μg ml^−1^ doxycycline. HEK293 and TM3 cells were grown according to instructions from American Type Culture Collection (www.atcc.org/). INS1E cells were cultured in RPMI containing 10 mM HEPES, 1 mM sodium pyruvate, 50 μg ml^−1^ β mercaptoethanol and 10% FBS. Rat islets were isolated by the standard collagenase digestion method from the pancreata of adult Sprague–Dawley rats (200–250 g) and cultured in RPMI medium (Invitrogen) with 10% FBS (Thermo Scientific). In brief, 9 ml of ice-cold Collagenase V (Sigma) solution was injected into the pancreas via the common bile duct. After dissection, the pancreas was incubated for 35 min at 37 °C and then further dissociated by repeated pipetting by using a 10-ml pipette. Islets were purified by Histopaque 1.077 (Sigma) density gradient centrifugation. Islets were allowed to recover from the isolation procedure for overnight in RPMI medium containing 10% FBS in non-tissue culture-treated petri dishes to prevent attachment. Human islets were obtained from Prodo Laboratories, Inc (Irvine, CA, USA), University of Minnesota and University of Miami, in accordance with internal review board (IRB) ethical guidelines for the use of human tissue. Typical viability was 80–90% and purity was>85%. Human islets were cultured in PIM-S medium (Prodo Laboratories, Inc.) containing 5% human AB serum. All cells were maintained at 37 °C, with 5% CO_2_ in a humidified atmosphere.

### Glucose stimulated insulin secretion from intact islets

Human islets were evaluated for glucose stimulated insulin secretion using standard methods. Secreted insulin was monitored using an ELISA (Meso-scale Devices) and normalized to total DNA (PicoGreen, Invitrogen).

### Rat and human islet proliferation assays

Rat or human β-cell proliferation assays were performed using dissociated primary rat or human islets. Islets were treated with the indicated compounds in the presence of EdU for 4 days before fixing and staining. EdU incorporation was measured by click reaction with AlexaFluor-647-azide (Invitrogen). For intact islet assays, primary human islets were treated for 7 or 8 days (as indicated in the legend). Rat dissociated islets were cultured in RPMI medium containing 10% FBS and human dissociated islets were cultured in PIM-S media (Prodo laboratories) with 10% human antibody serum.

### R7T1 proliferation assay

Reversibly immortalized mouse β-cells (R7T1)^49^ express SV40 T antigen under a tetracycline inducible promoter. R7T1 cells were expanded in the presence of doxycycline. For experiments on proliferation, R7T1 cells were growth arrested by removal of doxycycline for 2 days and plated into 384-well plates at a density of 3,000 cells per well in growth medium. β-Cell proliferation was assessed using CellTiter Glo (Promega) after 4 days. Fold increase in cell number was calculated by normalizing compound-treated wells to the median of DMSO-treated wells.

### Adenoviral overexpression of Gsk3b and Dyrk1a and NFATC1

Adenoviruses (Welgen) were diluted in the culture media, with a final concentrations (Dyrk1a WT 1:20 K; Gsk3b 1:20 K; NFATc1: 1:4,000–8,000; β-catenin adeno, 1:50 K) and added to dispersed rat islet cells in 384-well plates. Cells were incubated for 4 days with or without GNF7156 or GNF4877 treatment, fixed with 4% paraformaldehyde and EdU incorporation was measured by click reaction with AlexaFluor-647-azide (Invitrogen) and quantified similarly to the dispersed islet proliferation assay.

### β-Catenin and NFATc1 nuclear translocation

Dispersed rat islets were treated with GNF7156, GNF4877, GNF6324 or DMSO in 384-well plates according to the dispersed rat islet assay. At the end of the treatment, cells were fixed with 4% paraformaldehyde, blocked and permeabilized, and stained by standard immunofluorescence techniques for β-catenin (Cell signaling, #9562L; dilution 1:500), NFATc1 (Santa Cruz, rabbit polyclonal, SC-7294; dilution 1:200) or NFAT3c (Santa Cruz, SC-8321; dilution 1:200) and nuclear DNA was stained with DAPI (Molecular Probes; dilution 1:1,000). EdU incorporation was measured by click reaction with AlexaFluor-647-azide (Invitrogen). Plates were imaged with confocal microscope and analysed by Metamorph (Molecular Devices).

### Binding model of aminopyrazine compounds to Dyrk1a

Flexible docking was performed using Glide 5.8 (Schrodinger, Inc, Portland, OR, 2012). The protein coordinates were taken from protein databank (2vx3.pdb). The grid box was centred on the co-crystallized ligand and extended 10 Å from the centre, with outer box extending an additional 20 Å. The ligand was docked using the standard precision algorithm and scored using GlideScore.

### Single cell RNA sequencing

Whole islets from Sprague–Dawley rats were isolated followed by immediate hand picking to ensure removal of contaminating extra-islet cell populations. After isolation, whole islets were cultured in RPMI1640 (Gibco) containing 10% FBS and 5.5 mM glucose with either 0.1% DMSO or 3 μM GNF4877. Following 48 h of culture, islets were dissociated with 0.05% trypsin for 5 min at 37 °C into a single cell suspension and neutralized with media. Dispersed islets were passed through a 40 μm filter to remove un-dissociated cells and quantified by ViCell (Beckman Coulter).

Dispersed and quantified cells from islets were loaded into Fluidigm C1 IFC microfluidic chips according to manufacturer specifications (Fluidigm). Briefly, 60 μl of a 200 cell per μl mixture was combined with 40 μl of C1 suspension reagent. Five microlitre was loaded into the 10–17 μm Fluidigm IFC using the mRNA Seq: Cell Load (1,772x) protocol. After loading, the IFC was manually inspected by phase contrast microscopy to ensure single cell capture. Following inspection, lysis, reverse transcription, and PCR amplification were all performed on the IFC using the SMARTer Ultra Low RNA kit (Clontech) with the mRNA Seq: RT & Amp (1,772x) protocol. cDNA was quantified by PicoGreen assay (Life Technologies), normalized with C1 DNA dilution buffer to 0.1–0.3 ng μl^−1^, and sequencing libraries were prepared with the Nextera XT DNA Sample Preparation Index Kit (Illumina, PN FC-131-1002). Sequencing libraries were run on an Illumina HiSeq 1,000 sequencer using single 50 bp reads and one 96-well plate per lane. Of the 96 samples per IFC, 84 GNF4877-treated and 86 DMSO-treated samples containing greater than 100,000 mapped sequencing reads per sample and having a single verified cell per port were used for analysis. The average number of mapped sequencing reads was 1.26 million per cell. Reads were aligned to the mouse transcriptome using BWA (Li H. and Durbin R. (2009) Fast and accurate short read alignment with Burrows-Wheeler Transform, Bioinformatics, 25:1754-60. [PMID: 19451168]). Expression values are represented in reads per million.

To identify proliferating β-cells in GNF4877-treated islets, cells were assessed for *Cyclin B1* (*Ccnb1*) and *Insulin 1* (*Ins1*) gene co-expression. To uncover significant transcriptional changes between cell populations, the average of each gene's expression in proliferating β-cells of GNF4877-treated islets was divided by the average expression in β-cells of DMSO-treated islets (Ccnb1/DMSO). To ensure these changes were statistically significant, an unpaired Student's *t*-test was performed.

Gene ontology analysis was performed using DAVID (ref. 50) on those genes that had a Log2 fold change in Ccnb1 expressing β-cells of GNF4877 islets >1 with a *P* value of at least 0.05.

Volcano plot was generated using Spotfire (Tibco) and bivariate plots were generated in Prism (GraphPad).

## Additional information

**Accession codes:** Single Cell RNA-Seq data have been deposited in Gene Expression Omnibus under accession code GSE68625.

**How to cite this article**: Shen, W. *et al*. Inhibition of DYRK1A and GSK3B induces human β-cell proliferation. *Nat. Commun.* 6:8372 doi: 10.1038/ncomms9372 (2015).

## Supplementary Material

Supplementary Information Supplementary Figures 1-10, Supplementary Table 1 and Supplementary Methods
