# Supplementary material for: Inhibition of DYRK1A and GSK3B induces human β-cell proliferation
Source: Nat Commun. 2015 Oct 26;6:8372. doi: 10.1038/ncomms9372 (PMC4639830; doi:10.1038/ncomms9372)
Supplement: Supplementary Information — Supplementary Figures 1-10, Supplementary Table 1 and Supplementary Methods [file ncomms9372-s1.pdf]

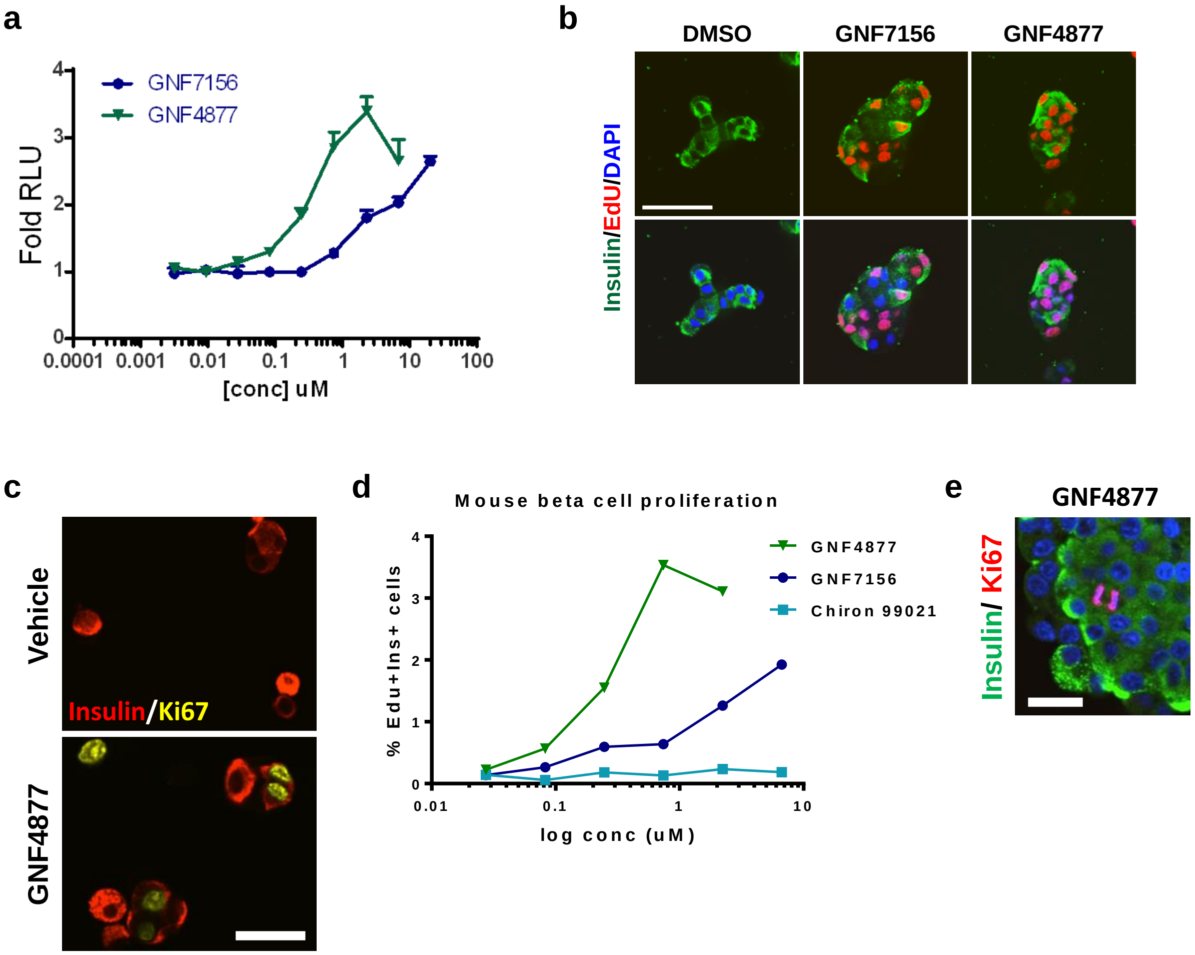

Supplementary Figure 1. AP compounds induce proliferation of β-cells. (a) Cell Titer-Glo assay after a 5 day incubation of growth-arrested R7T1 cells with AP analogs, and normalized to vehicle control (DMSO); data expressed as Relative luminescence unit (RLU); experimental treatments were performed in biological triplicates (mean ± SD; n=3). (b) Representative immunofluorescent images for Insulin and EdU of rat islets treated with GNF7156 or GNF4877 for 4 days (scale bar = 50 µm). (c) Images of human islets treated with vehicle or GNF4877 for 7 days and stained for insulin (red) and Ki67 (yellow) reveal a β-cell in cell division (scale bar = 25 µm). (d) Dose response of GNF7156, GNF4877 and Chiron99021-induced EdU incorporation into β-cells from dispersed mouse islets. Data are plotted as percent Insulin positive cells that are EdU positive (mean; n=3). (e) An example of an insulin positive cell from a primary adult human islet in anaphase. Intact islets were treated with GNF4877 for 7 days prior to staining for insulin, Ki67 and DAPI (scale bar = 20 µm).

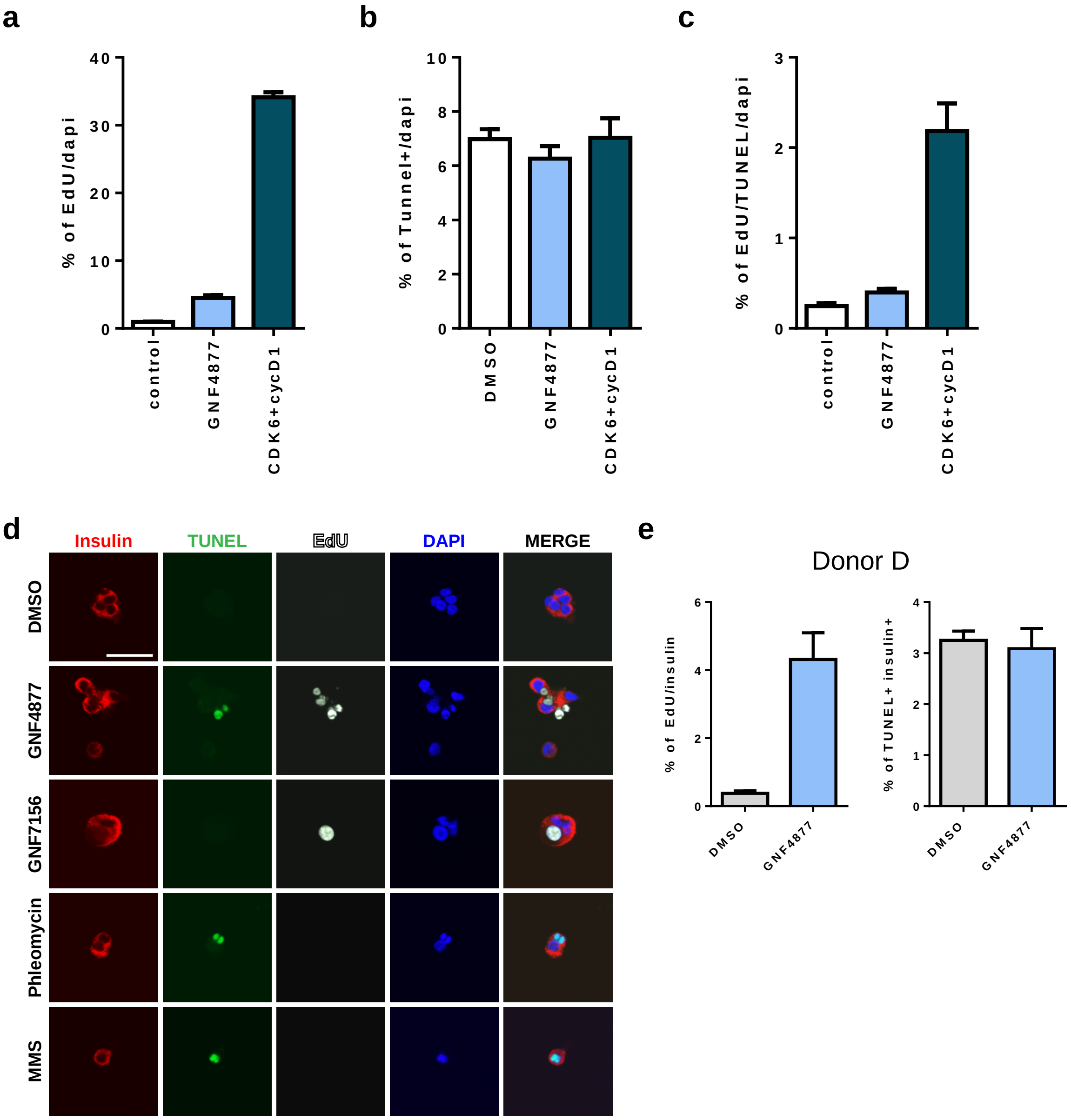

Supplementary Figure 2. AP compounds do not affect TUNEL in β-cells. (a-c) Primary human islets were treated with GNF4877 (2 µM) or infected with adenoviruses for CDK6 and Cyclin D1 for 4 days in the presence of EdU. GNF4877 increased EdU incorporation without significant effects on TUNEL in EdU positive cells while CDK6 and Cyclin D1 increased TUNEL staining in EdU positive cells (consistent with previous reports) (n=3; mean ± SD). (d) Human islets were treated with GNF4877, GNF7156 for 4 days; or Phleomycin or Methyl-methanesulfonate (MMS) for 16 hrs in the presence of EdU. Following treatment, cells were analyzed for TUNEL, and stained for insulin and DAPI. Confocal microscopy reveals that phleomycin and MMS induce TUNEL without EdU incorporation. GNF4877 and GNS7156 induce EdU incorporation; but do

not increase the percentage of TUNEL+ or TUNEL+/EdU+ cells. (e) Human islets from an additional donor were treated with GNF4877 for 4 days in the presence of EdU prior to staining and imaging. Increased EdU incorporation into insulin positive cells is observed while the percentage of TUNEL positive cells, insulin positive cells are unchanged by treatment with GNF4877 (n=3; mean ± SD).

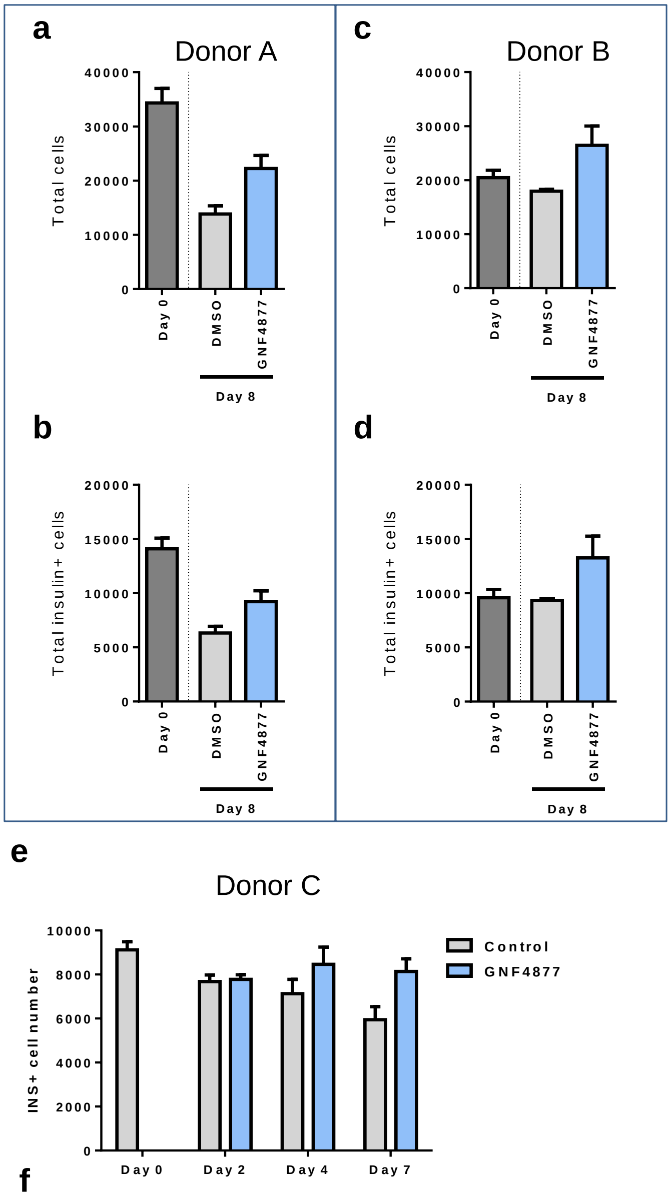

f

Donor	Age	Gender	BMI	HbA1c	Race
A	54	F	23.8	5.5%	White
B	44	M	27.1	5.3%	Latino
C	43	M	28.6	5.4%	White
D	51	M	31.2	5.1%	Asian

Supplementary Figure 3. GNF4877 increases primary adult human β-cell number relative to vehicle control. (a-d) Primary human islets from two different donors were treated with GNF4877 (2 μM) for 8 days prior to dissociation, staining and counting. Islets were taken prior to culture as the sample starting point (Day 0). (n=3; mean ± SD). (e) Primary human islets from

a third donor were treated with vehicle control or GNF4877 and samples were fixed at days 0, 2, 4 and 7 for quantifying total number of insulin positive cells. Progressive loss of insulin positive cells in the DMSO control islets is observed (consistent with previous reports). (f) Donor characteristics for the primary human islets used in these studies. (Donor D is in reference to the donor used in Supplemental Figure 2e).

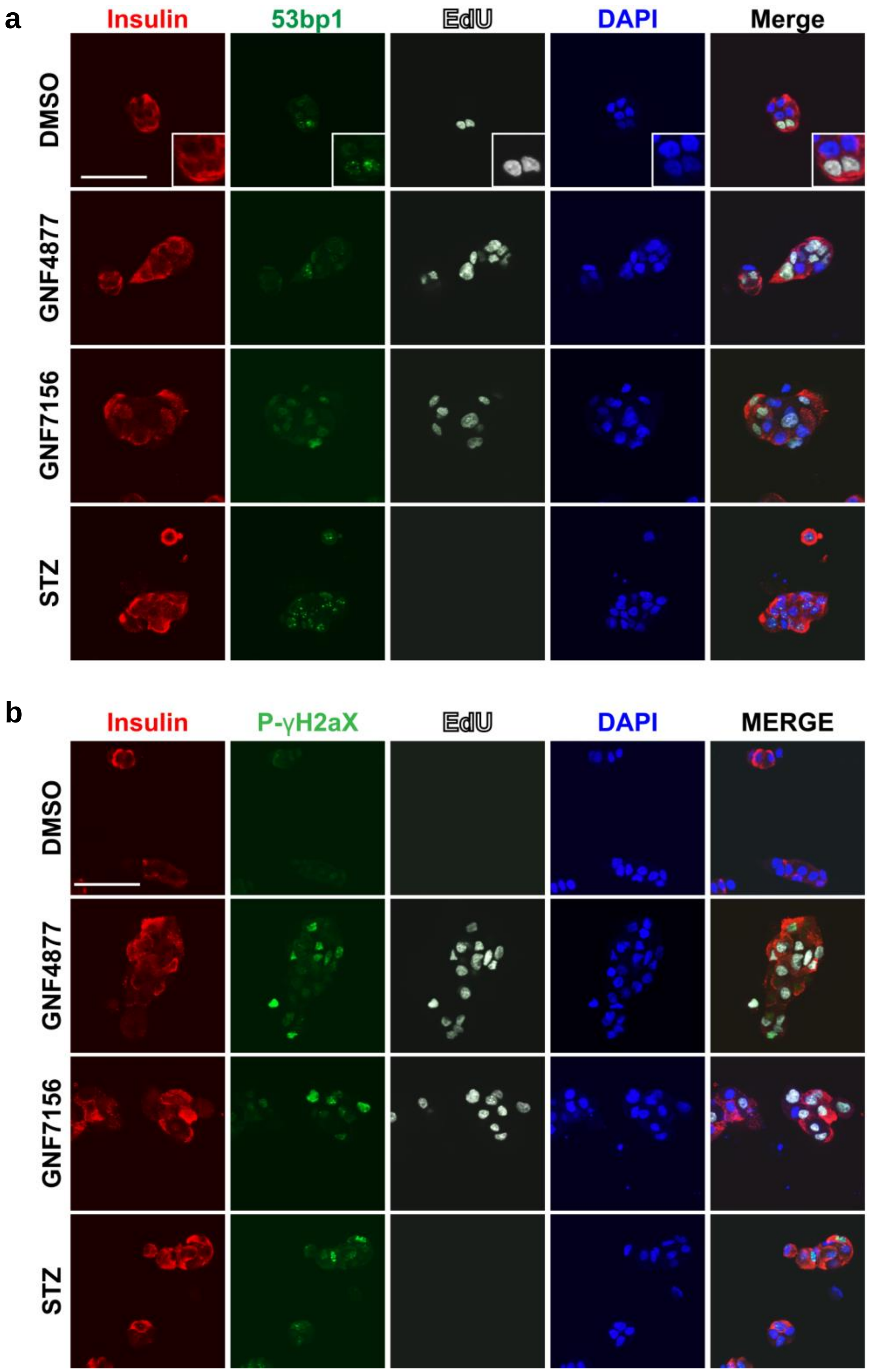

Supplementary Figure 4. AP compounds induce DNA damage markers in EdU positive rat β-cells. (a-b) Primary rat islets were treated with GNF4877, GNF7156 or STZ for four days prior to staining for insulin, EdU, p53 binding protein 1 (53BP1), and phospho-γH2aX. Imaging was performed by confocal microscopy. (a) All EdU positive cells displayed the presence of 53bp1

punctae, including in DMSO treated control islets (inset). STZ-induced DNA damage led to increased p53BP1 and phospho-γH2aX puncta without increased EdU incorporation. Scale bar = 100 μm.

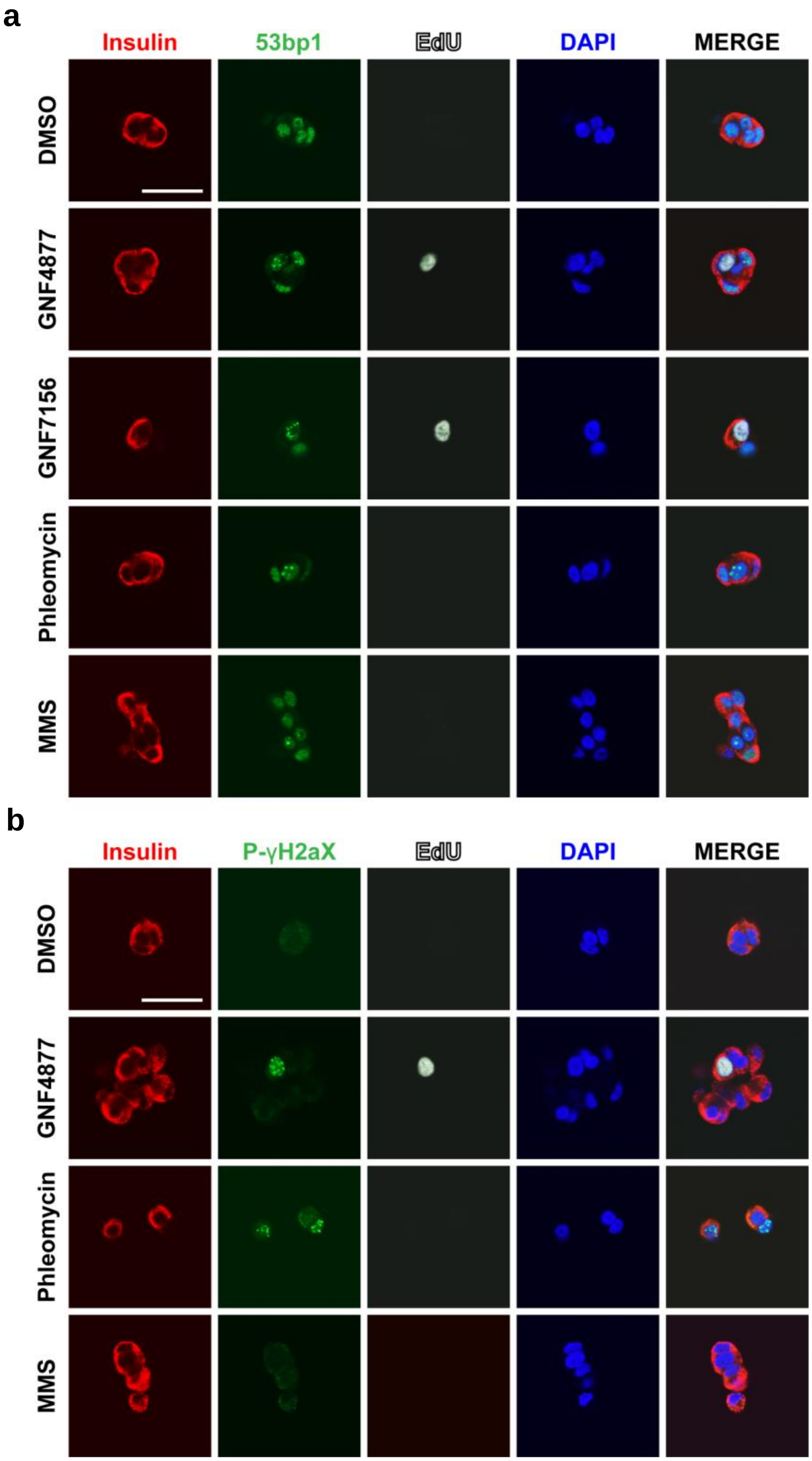

Supplementary Figure 5. AP compounds induce DNA damage makers in EdU positive β-cells of humans. (a-b) Primary human islets were treated with GNF4877, Phleomycin or MMS for four days prior to staining for insulin, EdU, p53 binding protein 1 (53BP1), and phospho-γH2aX.

Imaging was performed by confocal microscopy. DNA damaging agents led to increased p53BP1 and phospho-γH2aX puncta without increased EdU incorporation. By contrast, all EdU positive cells showed increased p53BP1 and γH2aX staining. Scale bar = 50 µm.

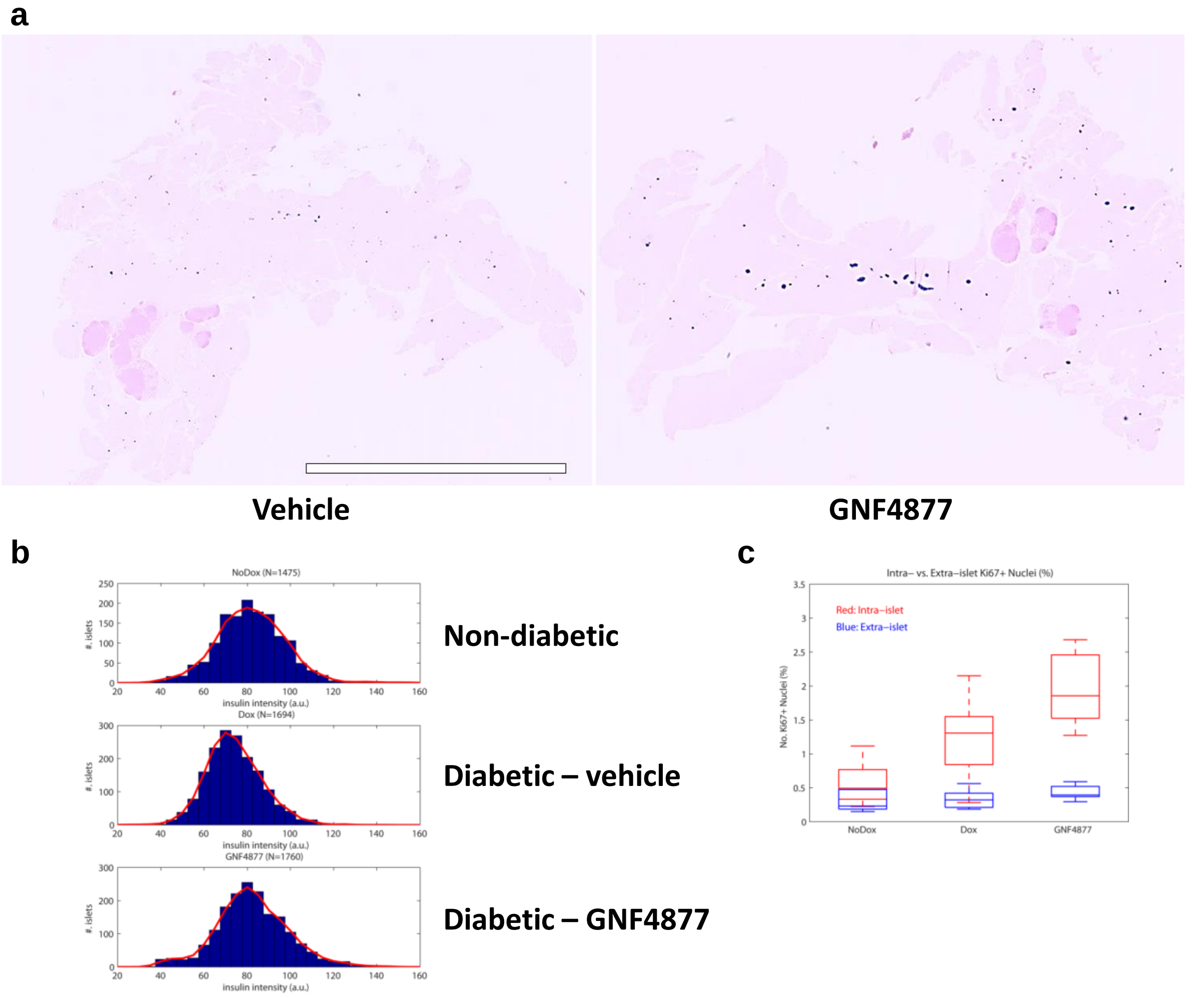

Supplementary Figure 6. GNF4877 increases beta cell mass in the RIP-DTA model of diabetes. (a-c) GNF4877 treatment leads to increased β-cell mass in diabetic RIP-DTA animals after treatment with GNF4877 for 14 days compared to vehicle control. (a) Insulin chromagen staining (dark blue) in pancreas sections counterstained with Nuclear Fast Red (pink). White bar represents 10 mm. (b) GNF4877 increases insulin immunofluorescent intensity relative to the diabetic vehicle control. Distribution of islet insulin intensity determined by automated β-cell mass calculation using MATLAB software. The red line is an estimate of the probability density based on the actual data. Number of islets counted was 1475 for non-diabetic, 1694 for vehicle-treated diabetic and 1760 for GNF4877-treated diabetic. (c) Comparison of intra-islet vs. extra-islet proliferation in pancreas sections by automated β-cell mass calculation using MATLAB software. GNF4877 increases intra-islet Ki67 positive cells without significantly increasing extra-islet Ki67. On each Box and whisker plot, the central mark is the median, the edges of the box are the 25th and 75th percentiles, and the whiskers extend to the most extreme data points.

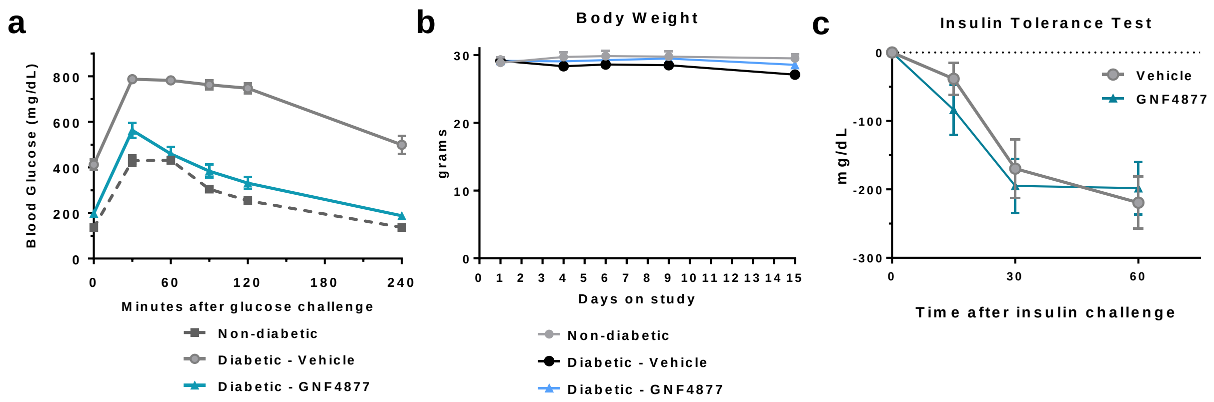

Supplementary Figure 7. Improved glucose tolerance without improved insulin tolerance in GNF4877-treated diabetic RIP-DTA mice. Diabetic RIP-DTA animals were treated for 16 days with GNF4877 or vehicle control (n=8 per treatment group). (a) GNF4877 improves glucose tolerance relative to vehicle-treated controls in diabetic RIP-DTA mice. After 16 days of treatment with GNF4877 (50 mg/kg, twice daily) or vehicle, animals were tested for oral glucose tolerance. Non-diabetic RIP-DTA mice were used for comparison. Data is shown as mean ± SD (n=8/group). (b) GNF4877 treatment (50 mg/kg, twice daily) did not alter body weight (mean ± SD; n=8 per group). (c) GNF4877 treatment of RIP-DTA mice does not improve insulin tolerance compared to vehicle-treated mice (mean ± SD; n=8 per group).

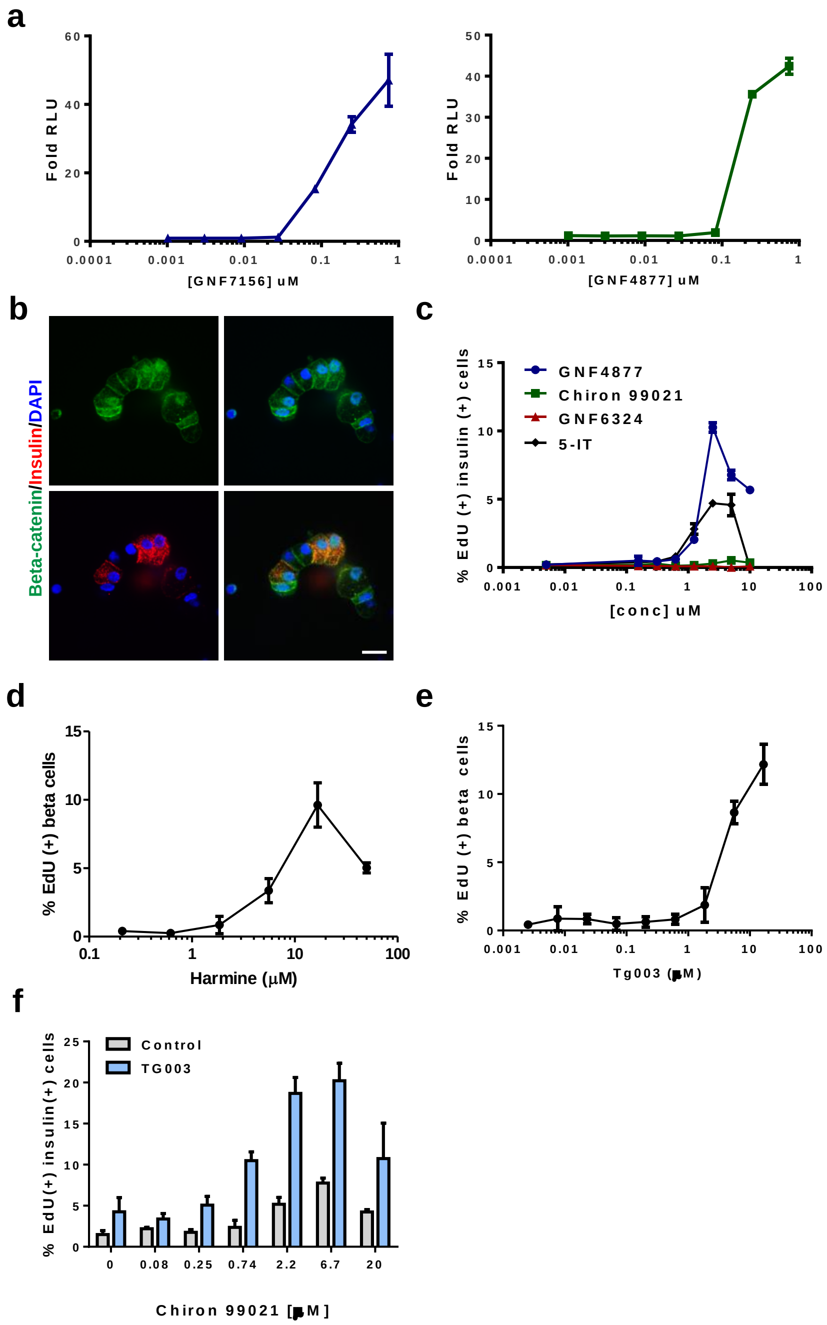

Supplementary Figure 8. Selective Dyrk1a inhibition is sufficient to induce β-cell proliferation. (a) GNF7156 and GNF4877 induce expression of a Wnt-driven luciferase reporter gene (Super Top Flash) in TM3 cells (mean ± SD; n=3). (b) GNF6324-treated primary rat islets were immunostained for nuclear localization of β-catenin in EdU and insulin co-positive cells. Co-localization of β-catenin with DAPI was observed in GNF6324-treated rat β-cells and not in the vehicle control cells. Scale bar = 20 μm. (c-e) Selective Dyrk1a inhibitors stimulate EdU

incorporation into primary human β-cells. Primary human islets were treated with the indicated compounds for 4 days and monitored for EdU incorporation into insulin positive cells by high content imaging. (c) Chiron 99021 and GNF6324 are selective GSK3β inhibitors, while 5-IT is a selective Dyrk1a inhibitor. Selective Dyrk1a inhibitors harmine (d) or Tg003 (e) stimulate primary human β-cell proliferation. (f) Combination of Dyrk1a and GSK3β inhibition lead to additive effects on EdU incorporation. Dissociated rat islets were treated with Chiron 99021 and TG003 (a selective Dyrk1a inhibitor) for 4 days. The percent of EdU positive cells was measured as a fraction of total insulin positive cells. In panels c-f, data is shown as mean ± SD (n=3).

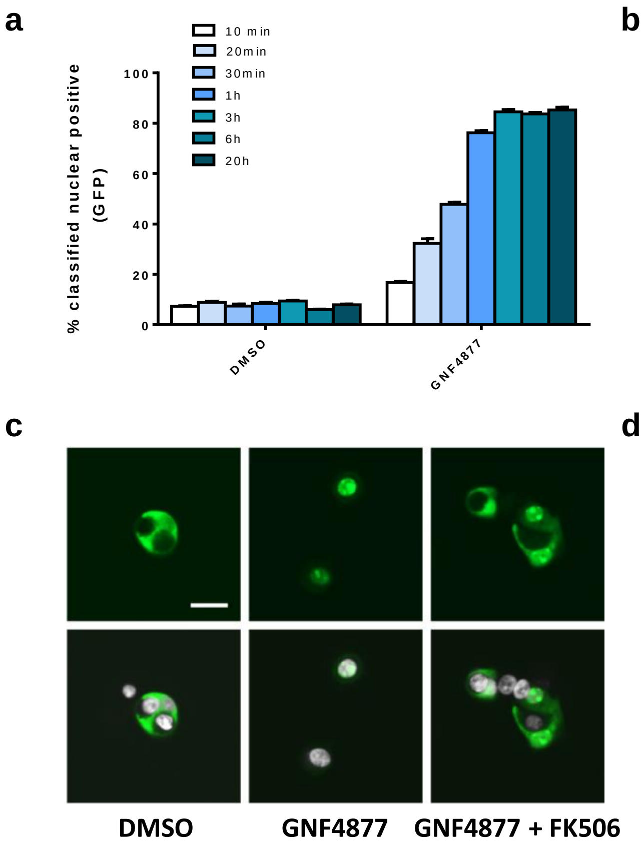

Supplementary Figure 9. AP compounds stimulate NFATc1 and NFATc2 nuclear localization. (a) Ins1 cells were infected with adenovirus expressing NFATc1-GFP and then treated with GNF4877 for the indicated time. Nuclear localization of NFATc1-GFP was quantified by high content imaging and data is shown as percentage of cells with nuclear NFATc1-GFP. The data suggest a slow accumulation of NFATc1 in the nucleus (mean +/- SD; n=3). (b) GNF4877 induces expression of an NFAT reporter gene assay in INS-1 cells. Ins1 cells were transfected with an NFAT driven reporter gene and luciferase activity was monitored. (c) GNF4877 induces NFATc1-GFP nuclear localization in primary human β-cells, which is partially abolished with co-treatment with a calcineurin inhibitor, FK506. NFATc1-GFP shown in green and DAPI in white. Scale bar = 25 µm. (d) Quantification of nuclear localization of NFATc2-GFP increases in response to GNF4877 or GNF7156 (mean +/- SD; n=3).

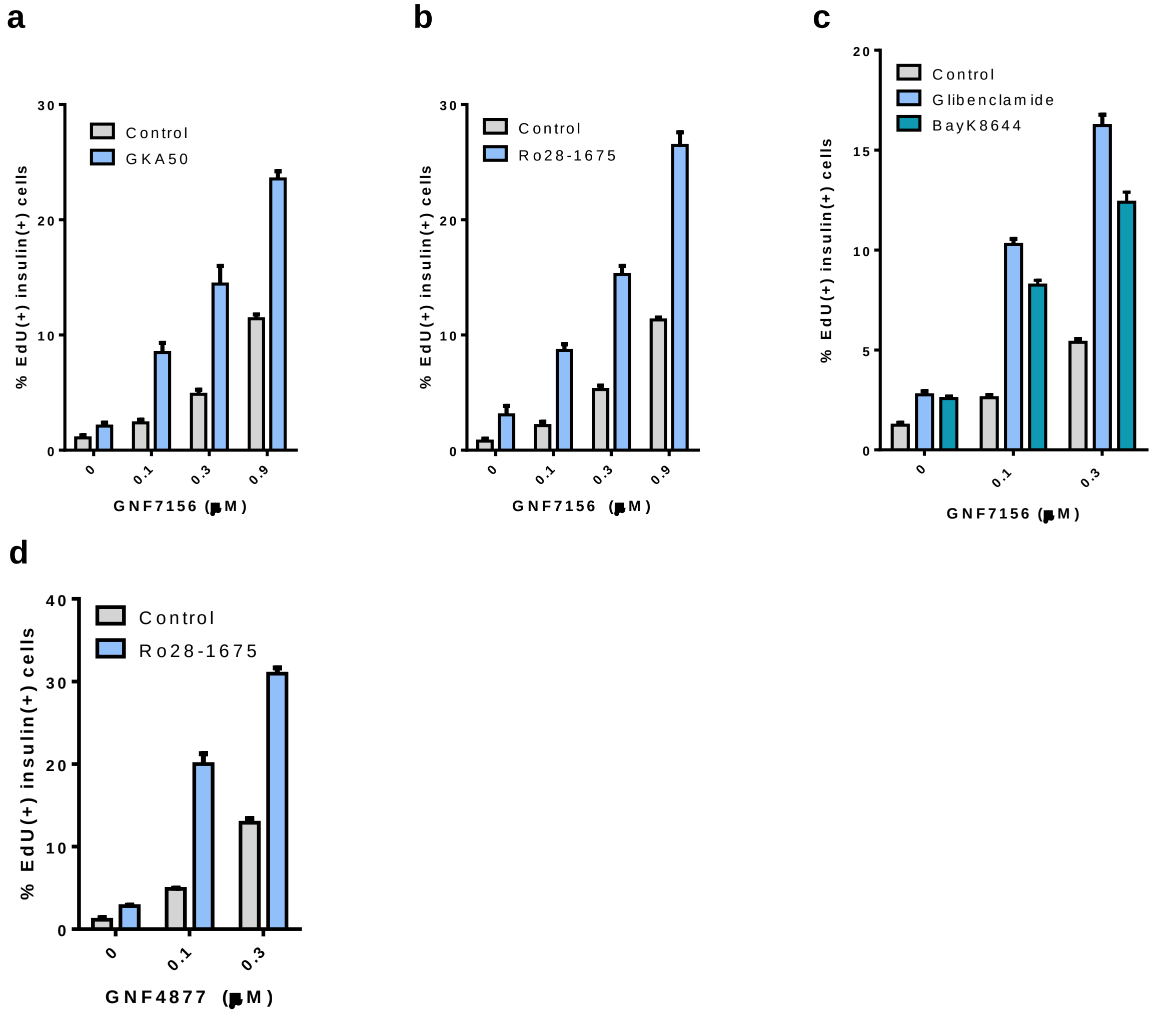

Supplementary Figure 10. Elevation of intracellular calcium levels shows synergy with AP compounds. (a-c) Concentrations of GNF7156 below the EC_{50} for rat β-cell proliferation are effective in inducing proliferation in the presence of agents that elevate intracellular calcium: (a-b) the glucokinase activators GKA50 and Ro28-1675; (c) a sulfonylurea receptor 1 inhibitor, glibenclamide, or an L-type Ca2+ channel activator, Bay K8644. (d) Concentrations of GNF4877 below the EC_{50} for rat β-cell proliferation are effective in inducing proliferation in the presence of the glucokinase activator Ro28-1675 (mean +/- SD; n=3).

Supplementary Table

Supplementary Table 1. Kinase profiling of GNF4877 and GNF7156.

	**Percent inhibition**		
**Column1**	**Column2**	**Column3**	**Column4**
Conc, nM	1000	1000	200
**Kinase**	**GNF7156**	**GNF4877**	**GNF4877**
Abl	90	66	
AKT3	7	25	
ALK	71	96	35
AURORA-A	95	84	0
AURORA-B	52	49	
Axl	88	94	74
BMX	88	43	
bRAF	46	20	
BTK	83	42	
CDK2/cyclinA	90	95	67
CHEK1	12	43	
CK1a	42	89	
c-Kit	49	77	
cRAF	17	18	
CSK	29	54	
DYRK1a	98	99	100
EGFR	71	44	
EphA3	82	63	
EphB3	12	28	
ErbB4 (HER4)	36	54	
FAK2 (PTK2)	92	70	9
FGFR3	60	32	
Flt-3	64	86	36
FMS (CSF1R)	78	72	
Fyn	83	77	26
GSK3beta	99	99	99
IGF1R	19	49	
IKKb	8	-1	
InsR	24	51	
IRAK4	25	52	
JAK2	92	95	2
JNK2	68	51	
KDR (VEGFR2, FLK1)	102	101	6

LCK	31	43	
LynA	56	54	
MAPK1 (ERK2)	6	7	
p38delta (MAPK13)	10	13	
p38alpha (MAPK14)	9	6	
MEK1	50	17	
MET	47	63	
NEK2	3	0	
p70S6K1	50	75	
PDGFRa	78	88	0
PDGFRb	94	94	0
PDK1	43	45	
PI3Ka	2	31	
PIM-2	60	31	
PKCalpha	96	97	0
PLK1	85	22	
RET	98	96	26
ROCK1	9	36	
ROS	96	97	71
SGK1	39	67	
SRC	61	64	
SYK	93	95	89
TRKA (NTRK1)	92	80	19
TTK	29	51	
TYRO3	52	46	
ZAP70	70	94	0

Supplementary Methods

Compound synthesis. Synthesis of GNF7156, GNF6324 and GNF4877

Synthesis of GNF7156:

Step 1: Methyl 3-amino-6-phenylpyrazine-2-carboxylate:

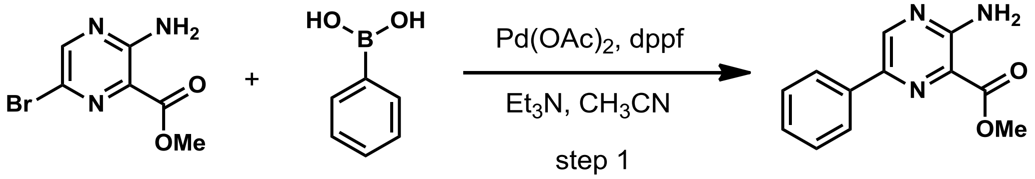

A stirring mixture of $Pd(OAc)_2$ (0.03 equiv.) and dppf (0.04 equiv.) in CH_3CN (0.02 M for dppf) was warmed to 50 ^{o}C and stirred for 30 min. After cooling to rt, methyl 3-amino-6-bromopyrazine-2-carboxylate (1.0 equiv.) and phenylboronic acid (1.2 equiv.) were added, and the reaction mixture was stirred for 3.5 hours at 100 ^{o}C in a sealed vessel. After the mixture was cooled to RT, water was added, and the mixture was extracted with DCM, washed with brine, and then dried over Na_2SO_4. The solvent was removed, and the residue was subjected to flash chromatography on silica gel (0-90% EtOAc/Hexanes eluent) to give the product as a yellow solid (75% yield). ^{1}H NMR ($CDCl_3$, 400 MHz): δ 8.68 (s, 1H), 7.93-7.89 (m, 2H), 7.50-7.44 (m, 2H), 7.42-7.36 (m, 1H), 6.47 (br, s, 2 H), 4.01 (s, 3H); ^{13}C NMR ($CDCl_3$, 125 MHz): δ 167.25, 154.61, 144.95, 142.45, 136.39, 129.05, 128.72, 126.05, 123.40, 52.87; LRMS (ESI) m/z 230.1 $[M+H]^{+}$.

Step 2: 3-amino-6-phenylpyrazine-2-carboxylic acid:

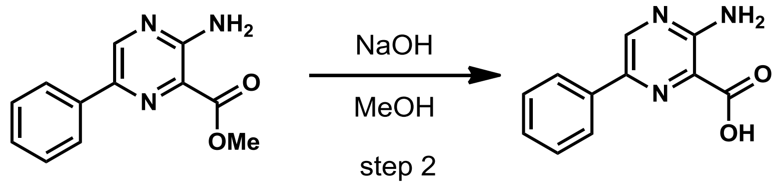

To a suspension of methyl 3-amino-6-phenylpyrazine-2-carboxylate (1.0 equiv.) in MeOH (0.5 M) was added NaOH (4 N, 2.5 equiv.). The reaction mixture was stirred for 3 hours at RT. More water was added, and the mixture was stirred for another 1 hour. Methanol was removed, and the residue was dissolved in water. The solution was washed with EtOAc once to get the clear solution, and then adjusted the pH to 2 by adding HCl. The solid precipitated was collected by filtration. ^{1}H NMR (CD_3OD, 400 MHz): δ 8.78 (s, 1H), 8.04-8.00 (m, 2H), 7.50-7.44 (m, 2H), 7.41-7.36 (m, 1H), 6.47 (br, s, 2 H); ^{13}C NMR (DMSO-d6, 125 MHz): δ 167.83, 154.89, 144.91, 139.36, 135.94, 128.70, 128.16, 125.39, 122.30; LRMS (ESI) m/z 216.1 $[M+H]^{+}$.

Step 3: Methyl 1-(3-(3-amino-6-phenylpyrazine-2-carboxamido)pyridin-4-yl)piperidine-4-carboxylate:

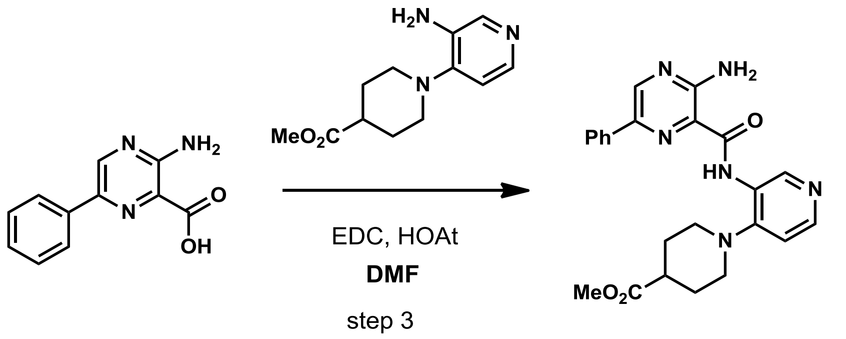

To a suspension of methyl 1-(3-aminopyridin-4-yl)piperidine-4-carboxylate (1.0 equiv.) and 3-amino-6-phenylpyrazine-2-carboxylic acid (1.2 equiv.) in DMF (0.2 M) was added EDC (1.2 equiv.) and HOAt (1.2 equiv.). The reaction mixture was stirred overnight at RT. DMF was removed under reduced pressure, and the residue was taken up into 20% i-PrOH in DCM, washed with water. The aqueous layer was back extracted with 20% i-PrOH/DCM twice. The combined organic layer was washed with brine, and then dried over Na_2SO_4. The solvent was removed, and the residue was subjected to flash chromatography on silica gel (0-60% EtOAc/DCM eluent) to give the product as a yellow solid (77% yield). ^{1}H NMR (DMSO-d6, 500 MHz): δ 10.36 (s, 1H), 9.37 (s, 1H), 8.95 (s, 1H), 8.29 (d, J = 5.0 Hz, 1H), 8.11-8.06 (m, 2H), 7.76 (br, s, 2H), 7.47-7.38 (m, 3H), 7.20 (d, J = 5.0 Hz, 1H), 3.31 (s, 3H), 3.16 (dt, J = 12.0, 3.5 Hz, 2H), 2.74 (td, J = 12.0, 2.5 Hz, 2H), 2.50-2.43 (m, 1H), 2.00-1.92 (m, 2H), 1.83-1.70 (m, 2H); ^{13}C NMR (DMSO-d6, 125 MHz): δ 173.84, 163.60, 154.37, 149.72, 145.92, 145.40, 141.21, 138.42, 135.36, 128.75, 128.28, 125.15, 122.87, 114.91, 51.38, 50.33, 27.94; LRMS (ESI) m/z 433.2 $[M+H]^+$.

Step 4: 1-(3-(3-amino-6-phenylpyrazine-2-carboxamido)pyridin-4-yl)piperidine-4-carboxylic acid (GNF7156):

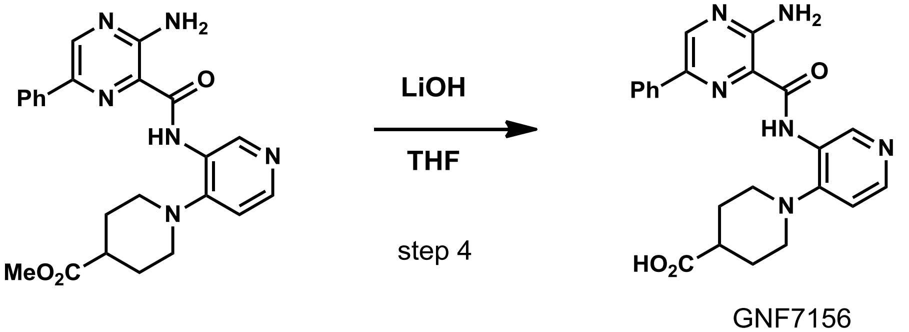

GNF7156

To a suspension of methyl 1-(3-(3-amino-6-phenylpyrazine-2-carboxamido)pyridin-4-yl)piperidine-4-carboxylate (1.0 equiv.) in THF-MeOH (2.5:1 v/v, 0.06M) was added LiOH (1M, 2.0 equiv.). The reaction mixture was stirred overnight at RT, and then acidified with HCl (1M, 2.0 equiv.). The organic solvent was removed under reduced pressure, and water was added. The solid was collected by filtration (98% yield). ^{1}H NMR (DMSO-d6, 400 MHz): δ 12.24 (s, 1H), 10.37 (s, 1H), 9.37 (s, 1H), 8.98 (s, 1H), 8.29 (d, J = 5.2 Hz, 1H), 8.11 (d, J = 7.6 Hz, 2H), 7.79 (br, s, 2H), 7.48 (t, J = 7.6 Hz, 2H), 7.38 (t, J = 7.6 Hz, 1H), 7.23 (d, J = 5.2 Hz, 1H), 3.21-3.15 (m, 2H), 2.81-2.72 (m, 2H), 2.44-2.31 (m, 1H), 2.01-1.91 (m, 2H), 1.85-1.72 (m, 2H); ^{13}C NMR (DMSO-d6, 125 MHz, compound was converted to HCl salt to improve the solubility): δ 175.17, 164.63, 154.65, 154.30, 145.34, 139.71, 139.29, 138.70, 135.48, 128.76, 128.36, 125.40, 124.21, 122.77, 113.55, 48.71, 27.75; LRMS (ESI) m/z 419.2 $[M+H]^+$; Anal. Caculated for C22H22N6O3: C, 63.15; H, 5.30; N, 20.08. Found: C, 62.83; H, 5.28; N, 19.97

Synthesis of GNF6324:

Step 1: methyl 1-(2-(3-amino-6-phenylpyrazine-2-carboxamido)phenyl)piperidine-4-carboxylate

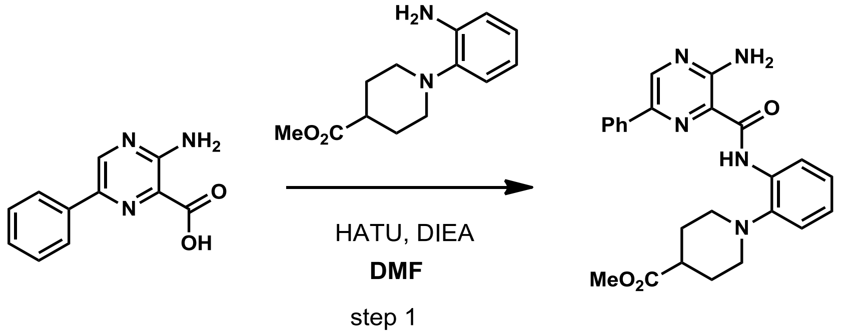

To a suspension of methyl 1-(3-aminopyridin-4-yl)piperidine-4-carboxylate (1.0 equiv.) and 3-amino-6-phenylpyrazine-2-carboxylic acid (1 equiv.) in DMF (0.2 M) was added HATU (1.2 equiv.) and DIEA (3 equiv.). The reaction mixture was stirred 3 hours at RT. DMF was removed under reduced pressure, and the residue was taken up into 20% i-PrOH in DCM, washed with water. The aqueous layer was back extracted with 20% i-PrOH/DCM twice. The combined organic layer was washed with brine, and then dried over Na_2SO_4. The solvent was removed, and the residue was subjected to flash chromatography on silica gel (0-5% MeOH/DCM eluent) to give the product as a biege solid (90% yield). ^{1}H NMR ($CDCl_3$, 500 MHz): δ 10.93 (s, 1H), 8.69 (s, 1H), 8.50 (dd, J = 8.5, 1.5 Hz, 1H), 8.02-7.97 (m, 1H), 7.51-7.45 (m, 2H), 7.43-7.38 (m, 1H), 7.22-7.17 (m, 2H), 7.24-7.09 (m, 1H), 3.59 (s, 3H), 3.16-3.07 (m, 2H), 2.79-2.71 (m, 2H), 2.43-2.35 (m, 1H), 2.09-2.03 (m, 2H), 1.99-1.89 (m, 2H); ^{13}C NMR ($CDCl_3$, 125 MHz): δ 174.74, 164.20,

154.31, 144.41, 143.21, 140.64, 136.11, 132.89, 129.07, 128.62, 125.78, 125.58, 125.15, 124.31, 120.34, 119.76, 52.52, 51.75, 40.49, 28.77; LRMS (ESI) m/z 432.2 $[M+H]^+$.

Step 2: 1-(2-(3-amino-6-phenylpyrazine-2-carboxamido)phenyl)piperidine-4-carboxylic acid (GNF6324)

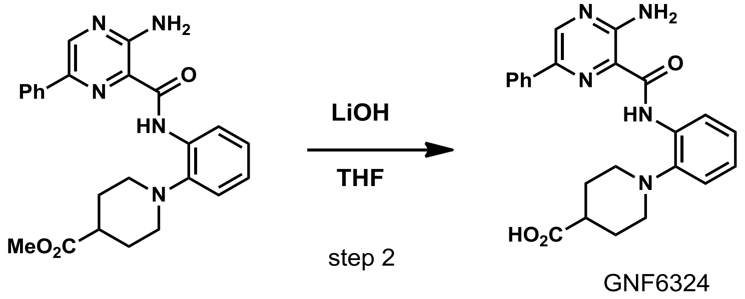

To a suspension of methyl 1-(2-(3-amino-6-phenylpyrazine-2-carboxamido)phenyl)piperidine-4-carboxylate (1.0 equiv.) in THF-MeOH (2.5:1 v/v, 0.06M) was added LiOH (1M, 6.0 equiv.). The reaction mixture was stirred overnight at RT. The mixture was diluted with water, washed with EtOAc once, and then acidified to pH = 3. The yellow solid precipitated was collected by filtration (78% yield). ^{1}H NMR (DMSO-d6, 400 MHz): δ 12.24 (s, 1H), 10.82 (s, 1H), 8.96 (s, 1H), 8.45 (dd, J = 8.0, 1.6 Hz, 1H), 8.11 (dd, J = 7.6, 1.2 Hz, 2H), 7.80 (br, s, 2H), 7.48 (t, J = 7.6 Hz, 2H), 7.38 (tt, J = 7.6, 1.2 Hz, 1H), 7.33 (dd, J = 7.6, 1.6 Hz, 1H), 7.18 (td, J = 7.6, 1.6 Hz, 1H), 7.13 (td, J = 7.6, 1.6 Hz, 1H), 3.05-2.95 (m, 2H), 2.80-2.70 (m, 2H), 2.43-2.31 (m, 1H), 2.05-1.93 (m, 2H), 1.90-1.77 (m, 2H); ^{13}C NMR (DMSO-d6, 125 MHz): δ 175.43, 163.78, 154.46, 145.06, 142.90, 138.38, 135.50, 132.50, 128.84, 128.29, 125.04, 124.70, 124.02, 123.47, 120.77, 118.72, 52.08, 28.42; LRMS (ESI) m/z 418.2 $[M+H]^+$.

Synthesis of GNF4877:

Step 1: (R)-ethyl 1-(3-nitropyridin-4-yl)piperidine-3-carboxylate (3):

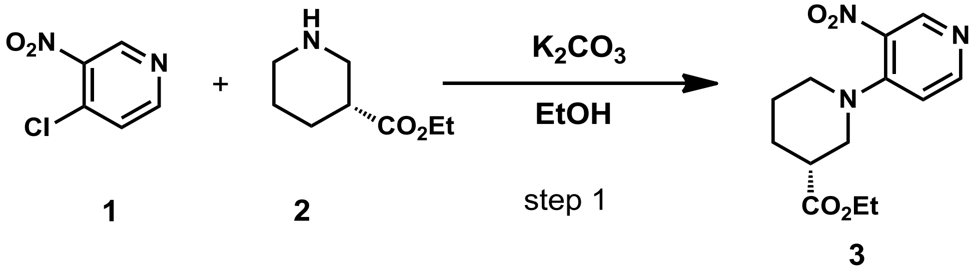

At 0 °C, to a suspension of 4-chloro-3-nitropyridine (1, 12.68 g, 80 mmol, 1.0 equiv.) in ethanol (100 mL) were added potassium carbonate (11.06 g, 80 mmol, 1.0 equiv.) and a solution of (R)-

ethyl piperidine-3-carboxylate (2, 12.58 g, 20 mmol, 1.0 equiv.) in ethanol (20 mL). The reaction mixture was stirred for 4.5h at 0 °C, then another 40 min at room temperature. The reaction mixture was diluted with water and extracted with DCM three times (3X100 mL). The combined organic layer was washed with brine, and then dried over anhydrous sodium sulfate. The solvent was removed, and the residue was purified on flash chromatography on silica gel (20-100% ethyl acetate/hexanes eluent) to afford the product as brown oil (20.98 g, 93% yields). Ee: 98.5% (Chiral Pak AD-H, CO2/MeOH 5% to 50% in 8 min, 2mL/min flow rate, temp 30 °C); ^{1}H NMR ($CDCl_3$, 500 MHz): δ 8.84 (s, 1H), 8.36 (d, J = 6.0 Hz, 1 H), 6.93 (d, J = 6.0 Hz, 1 H), 4.18-4.11 (m, 2H), 3.61-3.54 (m, 1 H), 3.34-3.27 (m, 1 H), 3.28 (dd, J = 13.0, 9.0 Hz, 1H), 3.08-3.00 (m, 1H), 2.76-2.68 (m, 1H), 2.18-2.09 (m, 1H), 1.87-1.67 (m, 3H), 1.24 (t, J = 7.0 Hz, 3H); ^{13}C NMR ($CDCl_3$, 125 MHz): δ 172.82, 152.76, 150.11, 148.46, 136.60, 113.40, 61.05, 51.72, 50.67, 41.03, 26.65, 23.97, 14.28; LRMS (ESI) m/z 280.1 $[M+H]^+$.

Step 2: (R)-ethyl 1-(3-aminopyridin-4-yl)piperidine-3-carboxylate (4):

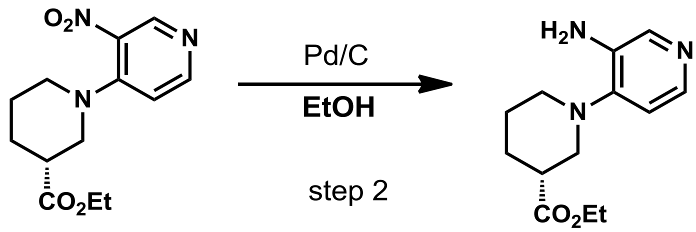

3 4

To a solution of (R)-ethyl 1-(3-nitropyridin-4-yl) piperidine-3-carboxylate (3, 12.11g, 1.0 equiv.) in ethyl acetate (40 mL) was added Pd/C (10% wt/wt, 1.21g, 0.026 equiv.). The mixture was shaken under H2 (50 psi) in a Parr hydrogenation flask for 3 days. The solid was filtered off, and the filtrate was concentrated to give the product was a yellow-brown oil (10.79 g). ^{1}H NMR ($CDCl_3$, 400 MHz): δ 8.01 (s, 1H), 7.94 (d, J = 5.2 Hz, 1 H), 6.79 (d, J = 5.2 Hz, 1 H), 4.26-4.14 (m, 2H), 4.07 (br s, 2H), 3.40-3.22 (m, 1H), 3.03-2.93 (m, 2H), 2.86-2.76 (m, 1H), 2.73-2.65 (m, 1H), 2.00-1.87 (m, 2H), 1.86-1.66 (m, 2H), 1.28 (t, J = 7.2 Hz, 3H); ^{13}C NMR ($CDCl_3$, 125 MHz): δ 173.98, 145.51, 140.76, 137.36, 137.26, 113.87, 60.77, 51.37, 50.48, 41.50, 26.58, 24.41, 14.28; LRMS (ESI) m/z 250.2 $[M+H]^+$.

Step 3: methyl 3-amino-6-(2-fluoro-5-isopropoxyphenyl)pyrazine-2-carboxylate (7)

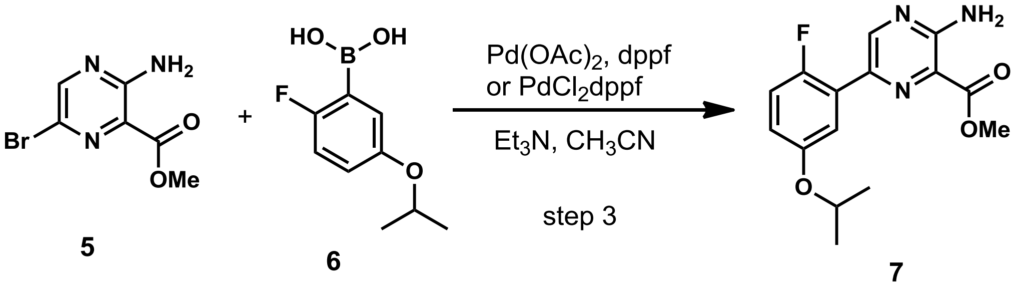

To a solution of (2-fluoro-5-isopropoxyphenyl)boronic acid (6, 950.4 mg, 4.8 mmol, 1.2 equiv.) in CH3CN (8.0 mL) was added methyl 3-amino-6-bromopyrazine-2-carboxylate (5,928.1 mg, 4.0 mmol, 1.0 equiv.), followed by PdCl2dppf (98.0 mg, 0.03 equiv.) and Et3N (0.89ml, 1.5 equiv.). The mixture was then heated at 100 °C for 4 hours in a sealed vial. After cooling to rt, the mixture was diluted with EtOAc, and washed with water, brine, and then dried over sodium sulfate. The solvent was removed, and the residue was subjected to flash chromatography on silica gel (0-40% EtOAc/hexanes eluent) to give the product (1144.2 mg, 94%). ^{1}H NMR ($CDCl_3$, 400 MHz): δ 8.70 (d, J = 2.4 Hz, 1H), 7.42 (dd, J = 6.4, 3.2 Hz, 1H), 7.05 (dd, J = 10.4, 9.2 Hz, 1H), 6.87-6.80 (m, 1H), 4.61 (Septet, J = 6.4 Hz, 1H), 4.00 (s, 3H), 1.34 (d, J = 6.4 Hz, 1H); ^{13}C NMR ($CDCl_3$, 125 MHz): δ 167.16, 154.73 (d, J_{CF} = 240 Hz), 154.58, 154.56 (d, J_{CF} = 2 Hz), 148.34 (d, J_{CF} = 14 Hz), 138.49 (d, J_{CF} = 4 Hz), 126.96 (d, J_{CF} = 14 Hz), 123.71, 117.59 (d, J_{CF} = 8 Hz), 117.16 (d, J_{CF} = 3 Hz), 116.87 (d, J_{CF} = 24 Hz), 70.93, 52.87, 22.19; ^{19}F NMR ($CDCl_3$, 500 MHz): δ -127.37; LRMS (ESI) m/z 306.2 $[M+H]^+$.

Step 4: 3-amino-6-(2-fluoro-5-isopropoxyphenyl)pyrazine-2-carboxylic acid (8)

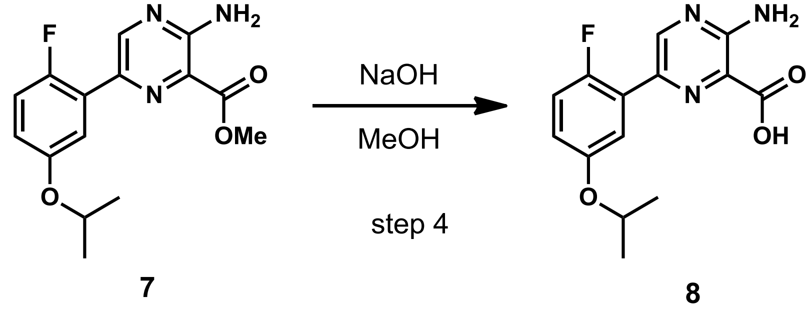

To a suspension of methyl 3-amino-6-(2-fluoro-5-isopropoxyphenyl) pyrazine-2-carboxylate (7, 1144.9 mg, 3.75 mmol, 1.0 equiv.) in MeOH (15 mL) was added NaOH (4 N, 2.34 mL, 2.5 equiv.). The reaction mixture was stirred for 3 hours at RT. Methanol was removed, and the residue was dissolved in water (~150 mL). The solution was washed with EtOAc once, and then adjusted the pH to 2 by adding HCl. The solid precipitated was collected by filtration (quantitative yield). ^{1}H NMR ($CDCl_3$, 500 MHz): δ 10.72 (br s, 1H), 8.80 (d, J = 1.0 Hz, 1H), 7.26 (dd, J = 6.5, 3.0 Hz, 1H),

7.10 (dd, J = 10.5, 9.0 Hz, 1H), 6.92 (ddd, J = 9.0, 4.0, 3.0 Hz, 1H), 4.54 (Septet, J = 6.0 Hz, 1H), 1.36 (d, J = 6.0 Hz, 1H); ^{13}C NMR (DMSO-d6, 125 MHz): δ 167.58, 154.74, 153.76 (d, J_{CF} = 238 Hz), 153.87, 147.45 (d, J_{CF} = 13 Hz), 135.42 (d, J_{CF} = 4 Hz), 124.53 (d, J_{CF} = 14 Hz), 123.13, 116.85 (d, J_{CF} = 25 Hz), 116.78 (d, J_{CF} = 7 Hz), 116.61 (d, J_{CF} = 3 Hz), 69.99, 21.77; ^{19}F NMR ($CDCl_3$, 500 MHz): δ -126.94; LRMS (ESI) m/z 292.1 $[M+H]^+$.

Step 5: (R)-ethyl 1-(3-(3-amino-6-(2-fluoro-5-isopropoxyphenyl)pyrazine-2-carboxamido)pyridin-4-yl)piperidine-3-carboxylate (9)

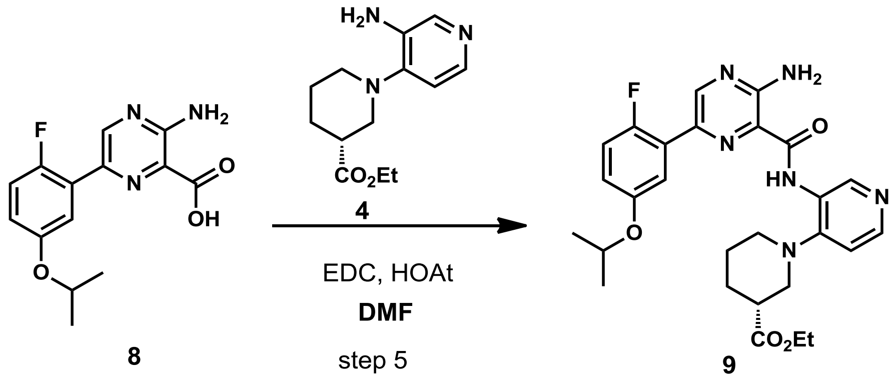

To a mixture of (R)-ethyl 1-(3-aminopyridin-4-yl) piperidine-3-carboxylate (4, 2.27 mmol, 565.9 mg, 1.0 equiv.) and 3-amino-6-(2-fluoro-5-isopropoxyphenyl)pyrazine-2-carboxylic acid (8, 2.73 mmol, 701.7 mg, 1.2 equiv.) in DMF (15.0 mL) was added EDC (523.3 mg, 1.2 equiv.) and HOAt (371.5 mg, 1.2 equiv.). The reaction mixture was stirred overnight at RT. DMF was removed under reduced pressure, and the residue was purified by reverse phase HPLC (10-70% MeCN(0.035% TFA)/water (0.05% TFA). After MeCN was removed under reduced pressure, the residue was extracted with 5% MeOH/DCM and organic layer was washed with saturated NaHCO3 (aq.) to yield 853.5 mg (1.63 mmol, 72%) compound 9 after removal of solvent. ^{1}H NMR (CD_3OD, 400 MHz): δ 9.45 (s, 1H), 8.65 (s, 1H), 8.23 (d, J = 5.6 Hz, 1H), 7.38-7.41 (m, 1H), 7.19 (d, J = 5.6 Hz, 1H), 7.14-7.16 (m, 1H), 6.97-4.01 (m, 1H), 4.61 (Septet, J = 6.4 Hz, 1H), 3.98-3.89 (m, 2H), 3.46-3.43(m, 1H), 3.24-3.21 (m, 1H), 2.80-2.70 (m, 2H), 2.71-2.63 (m, 1H), 1.89-1.84 (m, 1H), 1.80-1.74 (m, 2H), 1.48-1.38 (m, 1H), 1.33-1.31 (m, 6H), 1.07 (t, J = 7.2 Hz, 3H); ^{13}C NMR (CD_3OD, 100 MHz): δ 174.50, 165.15, 155.86, 155.70 (d, J_{CF} = 238 Hz), 152.12, 149.23 (d, J_{CF} = 10 Hz), 146.40, 142.05 (d, J_{CF} = 7 Hz), 137.27 (d, J_{CF} = 3 Hz), 130.16, 126.15 (d, J_{CF} = 14 Hz), 125.65, 118.97 (d, J_{CF} = 3 Hz), 117.80 (d, J_{CF} = 24 Hz), 117.13 (d, J_{CF} = 8 Hz), 116.29, 71.74, 61.51, 54.57, 52.17, 43.26, 27.52, 26.15, 22.34, 22.27, 14.30; ^{19}F NMR (CD_3OD, 500 MHz): -129.57; LRMS (ESI) m/z 523.30 $[M+H]^+$.

Step 6: (R)-1-(3-(3-amino-6-(2-fluoro-5-isopropoxyphenyl)pyrazine-2-carboxamido)pyridin-4-yl)piperidine-3-carboxylic acid (10, GNF4877)

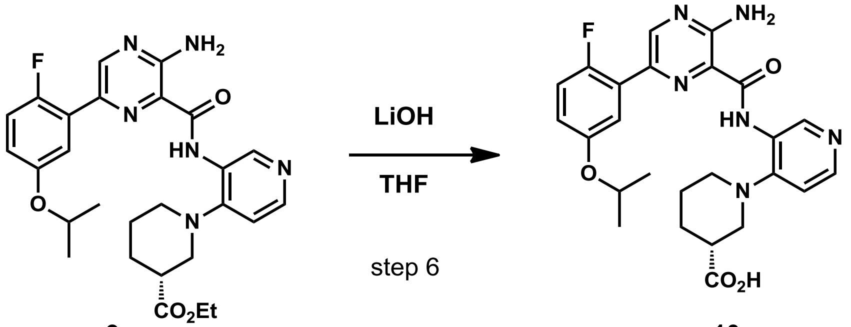

9 **10**

GNF4877

To a solution of compound 9 (522.6 mg, 1.0 mmol, 1.0 equiv.) in THF-MeOH (5.0 mL-3.0 mL) was added LiOH (1M, 2.0 equiv.). The reaction mixture was stirred for 4 hours at room temperature. The solvent was removed, and the residue was taken into water (~200 mL). The solution was washed with DCM once, and then acidified with HCl (1M, 2 equiv.). The solid precipitated was collected by filtration. After dried, yield 476.7 mg solid (96% yield). e.e.: 93.1% (Chiral HPLC Lux-Cellulose-1, 5 to 40% MeOH (0.5% DEA)/CO_2, flow rate 2mL/min, T_R = 9.29 (minor) and 9.63 min (major)) ; ^{1}H NMR (CD_3OD, 400 MHz): δ 9.46 (s, 1H), 8.64 (s, 1H), 8.20 (d, J = 5.6 Hz, 1H), 7.35-7.32 (m, 1H), 7.12 (d, J = 5.6 Hz, 1H), 7.12-7.07 (m, 1H), 6.92-6.88 (m, 1H), 4.53 (Septet, J = 6.0 Hz, 1H), 3.49-3.46 (m, 1H), 3.24-3.22 (m, 1H), 2.81-2.73 (m, 2H), 2.66-2.61 (m, 1H), 1.95-1.91 (m, 1H), 1.77-1.71 (m, 2H), 1.47-1.40 (m, 1H), 1.32-1.29 (m, 6H); ^{13}C NMR (DMSO-d6, 125 MHz): δ 174.22, 163.36, 154.24, 153.92, 153.61 (d, J_{CF} = 238 Hz), 149.64, 147.83 (d, J_{CF} = 10 Hz), 146.03, 141.50, 134.82 (d, J_{CF} =f 3 Hz), 127.95, 124.53 (d, J_{CF} = 14 Hz), 123.68, 117.17 (d, J_{CF} = 3 Hz), 116.80 (d, J_{CF} = 24 Hz), 115.84 (d, J_{CF} = 8 Hz), 114.92, 69.83, 52.78, 51.02, 41.31, 26.18, 24.51, 21.60, 21.59; ^{19}F NMR (DMSO-d6, 500 MHz): δ -127.59; LRMS (ESI) m/z 495.20 $[M+H]^+$; Anal. Caculated for C25H27FN6O4: C, 60.72; H, 5.50; N, 16.99. Found: C, 60.60; H, 5.46; N, 16.88.
